# Proposal, design, and cost analysis of a hydrogen production process from cellulose *via* supercritical water gasification†

**DOI:** 10.1039/d3ra05367a

**Published:** 2023-10-16

**Authors:** Taichi Masuda, Naoki Ikesaka, Yosuke Muranaka, Katsuaki Tanabe

**Affiliations:** a Department of Chemical Engineering, Kyoto University Nishikyo Kyoto 615-8510 Japan tanabe@cheme.kyoto-u.ac.jp

## Abstract

Hydrogen production from biomass, a renewable resource, has been attracting attention in recent years. We conduct a detailed process design for cellulose-derived hydrogen production *via* glucose using supercritical water gasification technology. Gasification of biomass in supercritical water offers advantages over conventional biomass conversion methods, including high gasification efficiency, elevated hydrogen molar fractions, and the minimization of drying process for wet biomass. In the process design, a continuous tank reactor is employed because the reaction in the glucose production process involves solids, and using a tube-type reactor may clog the reactor with solids. In the glucose separation process, glucose and levulinic acid, which cannot be separated by boiling point difference, are separated by using an extraction column. In the hydrogen separation process, the hydrogen purity, which could not be sufficiently increased with a single pressure swing adsorption (PSA) process, is increased to the target value by employing two sets of PSA columns. The overall utility cost is significantly reduced by $0.020/mol-H_2_ through heat integration. Our economic evaluation for this process results in a deficit of $0.015/mol-H_2_, as a price to be paid by the human for renewable hydrogen production from biomass at the present stage. By simply adopting the reported experimental condition, our process contains a large amount of water and sulfuric acid, which requires an enormous cost for the neutralizer, drying utility, and extractant. To improve the economic performance of the process, it is necessary to consider the reaction of cellulose solution at a higher concentration to reduce the burden of glucose separation. In addition, the effective use of the wasted hydrogen with a purity of about 95 vol% from the second PSA column may also improve the process economics. Whilst, the required energy cost for hydrogen production for our process is calculated to be significantly lower than those for other various representative hydrogen production methods: 0.37 (0.44) times less than that of steam reforming of methane with (without) CO_2_ capture, 0.15 times less than that of the water electrolysis by the electric power system, and 0.073 times less than that of electrolysis of water by wind power. This result implies the practical potential of our cellulose-based green hydrogen production scheme.

## Abstract 2

A cellulose-derived hydrogen production process *via* supercritical water gasification is designed. The required energy cost for our process is calculated significantly lower than those for other various representative hydrogen production methods.

## Graphical abstract

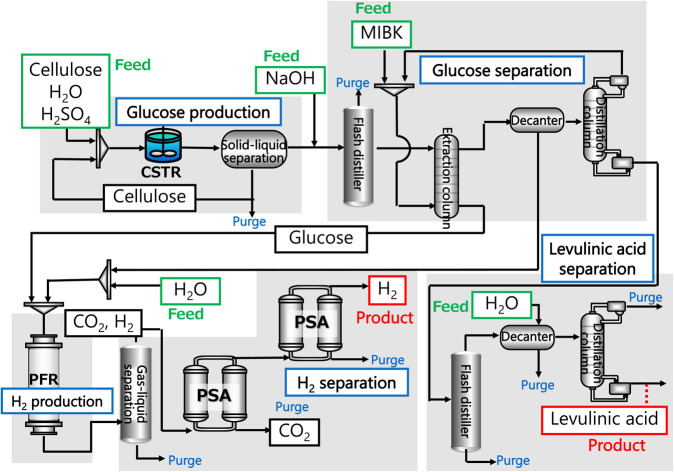

## 1. Introduction

Hydrogen energy is expected to expand its market significantly in the future as a next-generation energy source. However, 96% of the current hydrogen production is supplied by fossil fuels,^1,2^ which poses problems such as depletion of fossil fuel resources and acceleration of global warming due to carbon dioxide emissions. To address these problems, renewable hydrogen production methods are highly sought after.^3–9^ In this context, hydrogen production from biomass, a renewable resource, has been attracting attention in recent years. Biomass grows by capturing and condensing solar energy into chemical energy as carbohydrates through photosynthetic reactions of carbon dioxide and water. Therefore, the use of biomass as a fuel ensures carbon neutrality^10–15^ and benefits the environment. Nonetheless, conventional hydrogen production methods from biomass require dry feedstock, resulting in substantial energy consumption and cost for moisture drying.^16–20^ On the other hand, supercritical water gasification technology enables hydrogen production at high efficiency because the water content of biomass can be used as a solvent and reactant.^21–25^ This is because supercritical water gas has very different properties from liquid water: the dielectric constant of supercritical water is significantly lower, the number of hydrogen bonds is significantly smaller, and their bond strength is significantly weaker. Consequently, supercritical water behaves like many organic solvents, and organic compounds are completely miscible with supercritical water. Furthermore, supercritical water allows chemical reactions to take place in a single fluid phase because gases are also miscible with supercritical water, whereas reactions occur in multiphase systems under conventional environments. Therefore, gasification of biomass in supercritical water offers numerous advantages over other biomass conversion methods, including high gasification efficiency, elevated hydrogen molar fractions, and the elimination of a drying process for wet biomass.^21–25^ In the present study, we propose and conduct a detailed process design for cellulose-derived hydrogen production using supercritical water gasification technology. Cellulose stands out as a superior biomass material for hydrogen production, particularly when compared to other biomass sources. Its widespread availability from agricultural residues, forest waste, and dedicated energy crops positions cellulose as a highly abundant and renewable resource that minimizes concerns about food competition. Unlike lignocellulosic biomass, which requires complex processing due to its mixture of cellulose, hemicellulose, and lignin, cellulose offers a streamlined feedstock for hydrogen production. Its high hydrogen content and compatibility with efficient gasification and fermentation processes make cellulose an optimal choice. Algae, while fast-growing and lipid-rich, face challenges in energy-intensive harvesting and lipid extraction. Moreover, cellulose's well-established conversion technologies and ongoing research in catalysts and reactors contribute to its status as a prime candidate for scalable, sustainable, and cost-effective hydrogen production, ultimately driving the advancement of a cleaner energy future.

## 2. Methods

### 2.1. Overview of the entire process and operation conditions

**Fig. 1:**
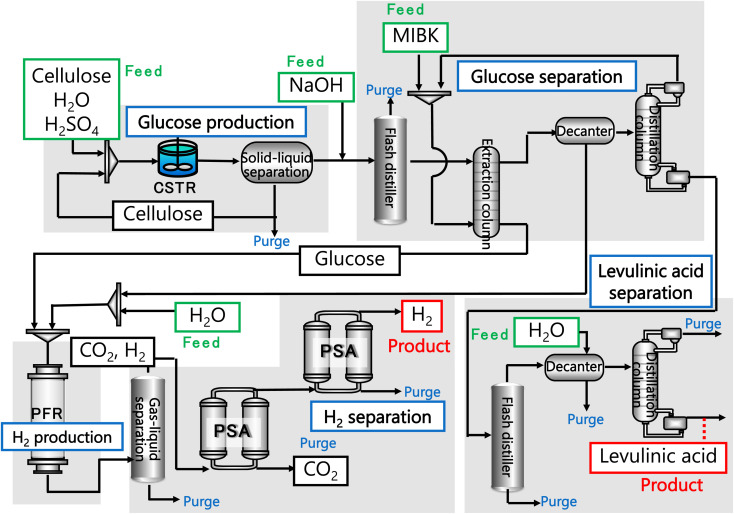
Schematic flow diagram of the entire process. CSTR is continuous stirred-tank reactor, MIBK is methyl isobutyl ketone, PFR is plug flow reactor, and PSA is pressure swing adsorption.

**Fig. 2:**
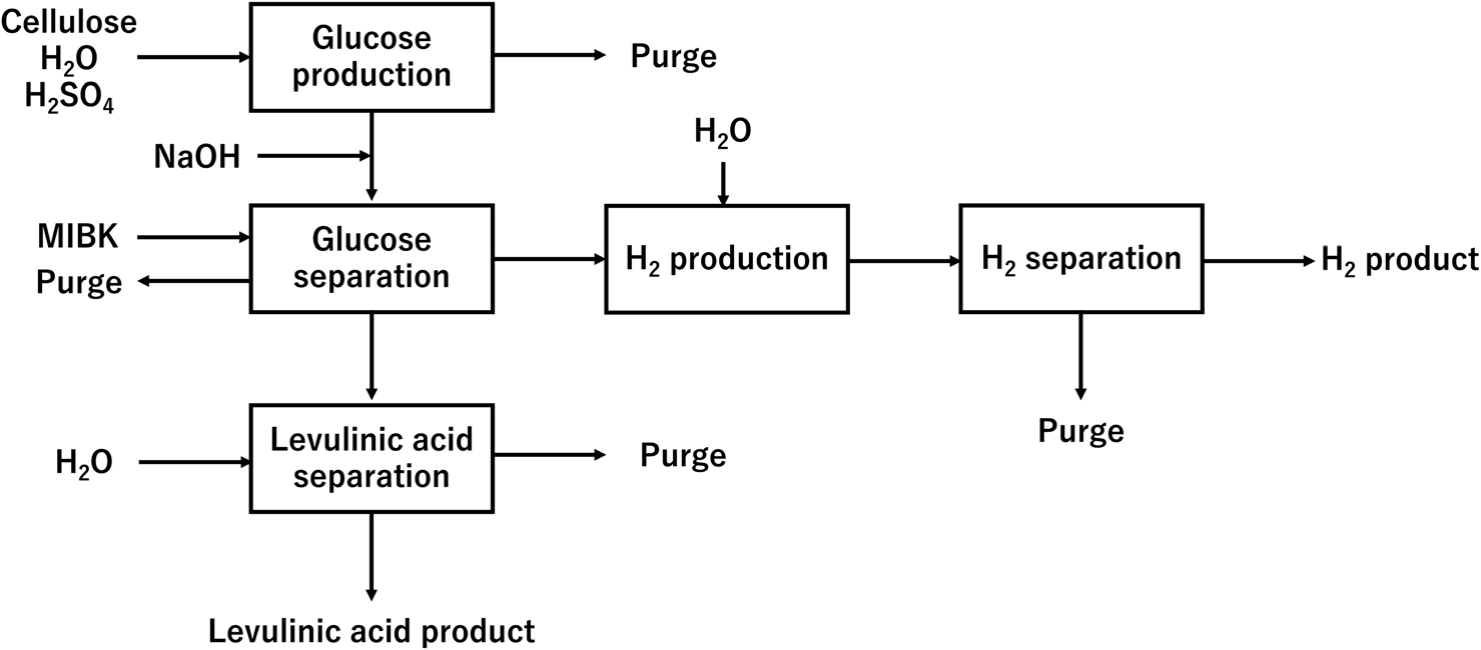
Block flow diagram of the entire process. MIBK is methyl isobutyl ketone.

In our process, glucose and levulinic acid are produced by the degradation reaction of cellulose, and hydrogen is produced from glucose by the supercritical water gasification reaction. Fig. 1 and 2 show the schematic and block flow diagrams of the entire process. Cellulose is fed to the process with dilute sulfuric acid, and glucose and levulinic acid are produced in the glucose production process. After neutralization with sodium hydroxide, glucose is extracted in the glucose separation process. The extracted glucose is reacted with supercritical water in the hydrogen production process to produce hydrogen. The purity of hydrogen and levulinic acid is increased by the hydrogen separation process and the levulinic acid separation process, respectively, and these products are commercialized. Numerical process simulators Aspen Plus and Aspen HYSYS (Aspen Technology Inc.) were used to compute the mass and heat balances in the reaction and separation processes.

The process operation conditions are as follows. We design a plant to produce 41 million Nm^3^ per year of 99.99 vol% pure hydrogen. The plant will also produce 99 wt% pure levulinic acid as a co-product. The plant will operate for 8000 hours per year. The raw material will be cellulose and dilute sulfuric acid in a ratio of 4 : 96 (by mass) at a pressure of 1 bar and a temperature of 25 °C. The purities of chemicals, including water, supplied to the process are assumed to be 100%. The concentration of dilute sulfuric acid is 0.3 mol L^−1^ as a result of optimization as described later.

### 2.2. Glucose production process

The glucose production process is degrades cellulose and is a solid–liquid reaction in which cellulose, glucose and humin are solids and all other substances are liquids. The minimum pressure at which all substances except cellulose and humin become liquids was 16 bar in the Aspen HYSYS simulation, so the pressure in the reactor was set to 16 bar. The involved reactions and their rates are:^26^2-2-1
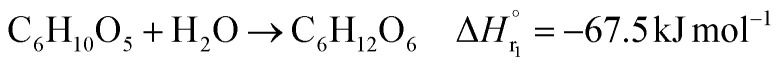
2-2-2
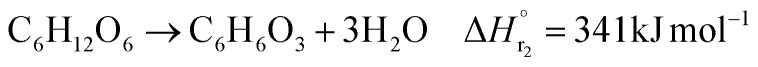
2-2-3
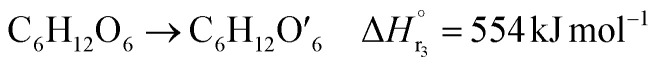
2-2-4
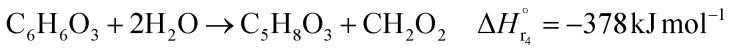
2-2-5
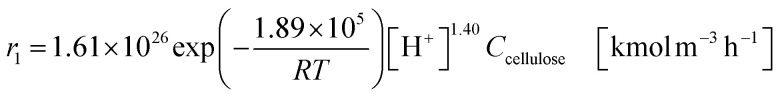
2-2-6
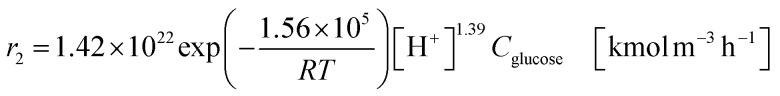
2-2-7
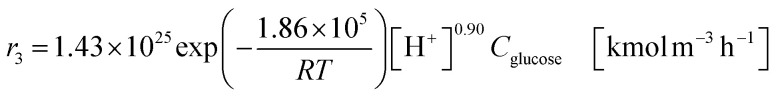
2-2-8
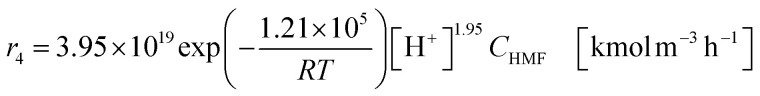


(C_6_H_10_O_5_: cellulose, C_6_H_12_O_6_: glucose, C_6_H_6_O_3_: hydroxymethylfurfural (HMF), 
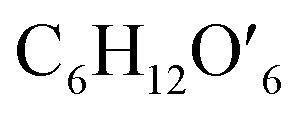
: humin, C_5_H_8_O_3_: levulinic acid (LA), CH_2_O_2_: formic acid (FA), *r*_*i*_: each reaction or reaction rate, 
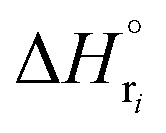
: standard reaction enthalpy of each reaction, *R* [J mol^−1^ K^−1^]: gas constant, *T* [K]: reaction temperature, [H^+^] [mol L^−1^]: hydrogen ion concentration, *C*_cellulose_ [kmol m^−3^]: cellulose concentration, *C*_glucose_ [kmol m^−3^]: glucose concentration, *C*_HMF_ [kmol m^−3^]: HMF concentration). For thermal calculations, the temperature-dependent molar heat capacity at constant pressure of each chemical substance reported in ref. 27 and 28 and those extracted from Aspen Plus, and the standard formation enthalpy values in ref. 27–30 were employed.

Because this glucose production reaction is a solid–liquid reaction in which cellulose, glucose, and humin are solids and other substances are liquids, a continuous stirred-tank reactor (CSTR) was employed to circumvent the potential clogging of the solids in a tube reactor. As we numerically tested simulating the temperature evolution in the reactor with a condition of the inlet temperature of 200 °C, the number of tanks of five, the residence time of 13 s, and the sulfuric-acid concentration of 0.3 mol L^−1^, the maximum decrease of temperature was found to be as small as 5.4 °C even for the highest glucose yield of 0.40. Therefore, the reaction was to operate under adiabatic conditions. In addition, since this reaction is performed under high-temperature dilute sulfuric acid conditions, a Ni alloy called Alloy20, which is resistant to sulfuric acid, was employed as the reactor material.

In the entire glucose production reaction, there are four elementary reactions and thus independent stoichiometric equations, and seven components whose amount of substance changes with the reaction, as seen in eqn (2-2-1)–(2-2-4). Cellulose, glucose, HMF, and levulinic acid were selected as key components, for which the mass and heat balance can be formulated as:

2-2-9 *F*_cellulose0_ − *F*_cellulose_ − *r*_1_*V* = 0

2-2-10 *F*_glucose0_ − *F*_glucose_ + (*r*_1_ − *r*_2_ − *r*_3_) *V* = 0

2-2-11 *F*_HMF0_ − *F*_HMF_ + (*r*_2_ − *r*_4_) *V* = 0

2-2-12 *F*_LA0_ − *F*_LA_ + *r*_4_*V* = 0

2-2-13
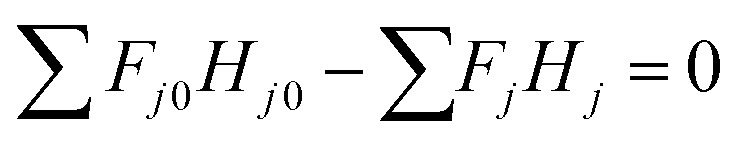


(*F*_*j*0_ [kmol h^−1^]: molar flow rate of the component *j* at the reactor inlet, *F*_*j*_ [kmol h^−1^]: molar flow rate of the component *j* at the reactor outlet, *r*_*i*_ [kmol m^−3^ h^−1^]: reaction rate of the reaction *i*, *V* [m^3^]: reactor volume, LA: levulinic acid, *H*_*j*0_ [kJ kmol^−1^]: enthalpy of the component *j* at the reactor inlet, *H*_*j*_ [kJ kmol^−1^]: enthalpy of the component *j* at the reactor outlet). The molar concentration of the key components and temperature at the reactor outlet were calculated by solving nonlinear simultaneous equations using the Newton method for multivariable functions, by taking the molar concentration of the key components and temperature at the reactor inlet as variables. The following assumptions were employed in our calculations: the volume flow rate is always constant; the fluid is a perfect mixture; pressure drop is neglected; glucose is dissolved in water; cellulose and humin do not aggregate and the solution is well stirred. By referring ref. 1, the design conditions were set as follows: the concentration of supplied cellulose solution of 4 wt%, the reactor pressure of 16 bar, the reactor temperature between 150 and 200 °C, and the sulfuric-acid concentration between 0.1 and 0.526 mol L^−1^. The reactor outlet fluid was assumed to be capable of separating cellulose and humin at 10% water content by solid–liquid separation. The operating power was assumed to be 0.6 kW, referring to vacuum drum filters.^31^ The recovered cellulose and humin were recycled to the reactor inlet at a purge rate of 5%. Optimization of the process conditions was performed to minimize the sum of construction cost, utility cost, and raw material cost for the reactor and heat exchanger by taking the inlet temperature, number of tanks, residence time, and sulfuric acid concentration as variables. In addition, the volumes of tanks were considered to be equal to one another.

### 2.3. Glucose separation process

**Fig. 3:**
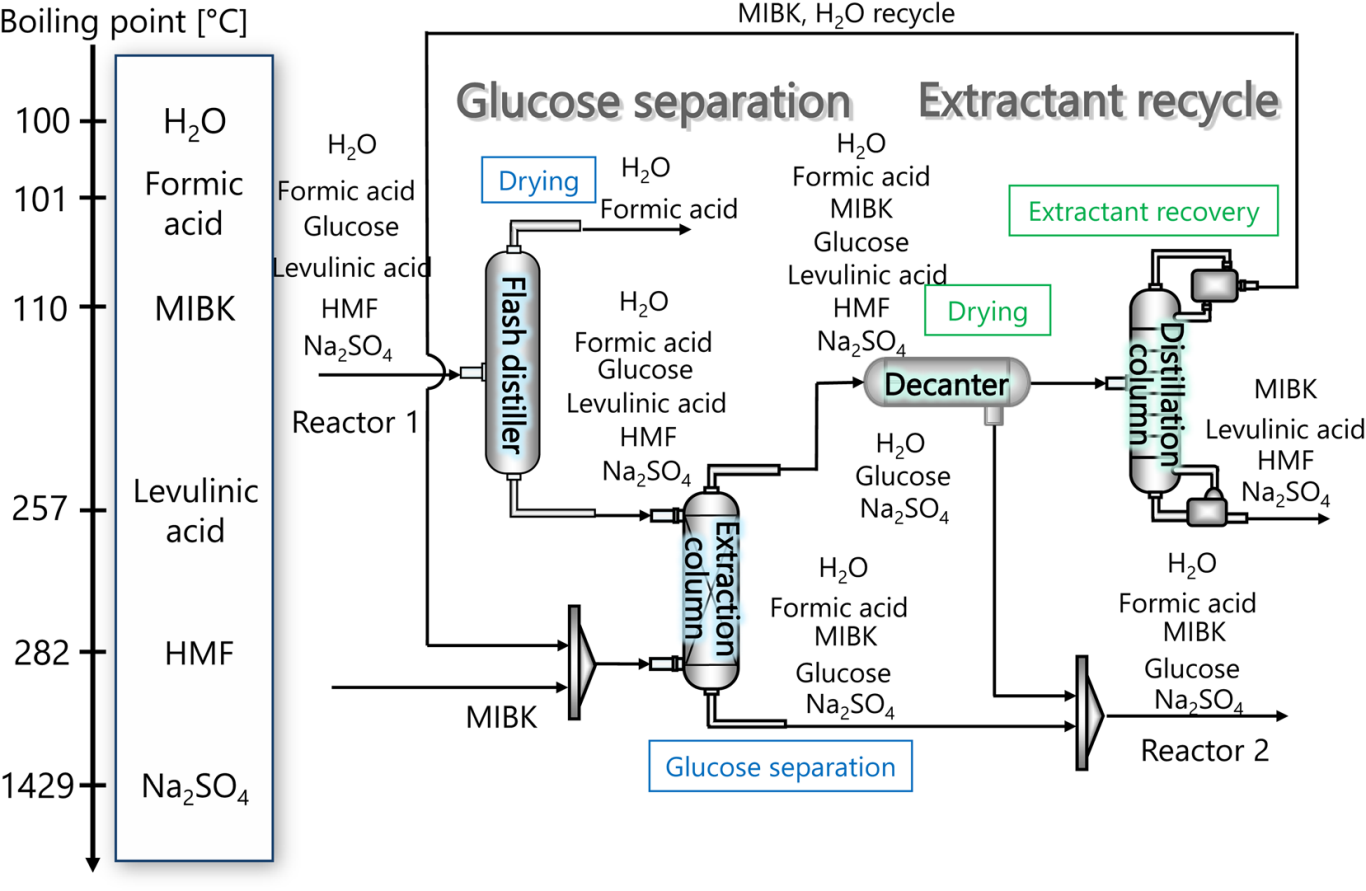
Flow diagram of the glucose separation process, along with the boiling points of the involved components. Note that glucose has no boiling point because it thermally decomposes above its melting point, 150 °C.^32^ MIBK is methyl isobutyl ketone and HMF is hydroxymethylfurfural.

The glucose separation process involves two treatments of the content from the glucose production process. The first is to remove the excess water for the subsequent hydrogen production process. The second is to separate HMF and levulinic acid from the content. Fig. 3 presents the flow diagram of the glucose separation process and the boiling points of the involved components. Note that glucose has no boiling point because it thermally decomposes above its melting point, 150 °C.^32^ The flash distiller separates the gaseous H_2_O and formic acid. In the extraction column, glucose in the aqueous phase is separated from levulinic acid and HMF in the oil phase. The oil phase components then flow into the decanter, where the water and glucose in the oil phase are separated. The oil phase component from the decanter then flows into the distillation column, where methyl isobutyl ketone (MIBK) is recovered from the top of the column and recycled. Meanwhile, Na_2_SO_4_, HMF and levulinic acid are taken out from the bottom of the column and sent to the levulinic acid separation process. The non-random two-liquid (NRTL) model^33^ was used for the physical property estimation equations. However, as the parameters for the two-component interactions of levulinic acid and glucose, glucose and formic acid, MIBK and glucose, levulinic acid and formic acid, MIBK and levulinic acid, and MIBK and formic acid do not exist as data in Aspen Plus, the universal functional group activity coefficient (UNIFAC) method^34^ was used to estimate the parameters of the two-component interactions. For the parameters of the two-component interactions of HMF with water and MIBK with HMF, the values from ref. 35 were used. Since high temperatures in the glucose separation process under dilute sulfuric acid conditions may cause the same reaction as in the glucose production process, neutralization with sodium hydroxide is performed before the glucose separation process. In the neutralization, the heat of neutralization was considered to be generated according to the following equation.

2-3-1 H_2_SO_4_(aq) + 2NaOH(aq) → Na_2_SO_4_(aq) + 2H_2_O(l) Δ*H* = −114.6 kJ mol^−1^

Fig. 3 flow diagram of the glucose separation process, along with the boiling points of the involved components. Note that glucose has no boiling point because it thermally decomposes above its melting point, 150 °C.^32^

The flash distiller separates water that is unnecessary in the hydrogen production process. Therefore, a design condition was set that the flow rate of water at the outlet should be less than the flow rate of water required for hydrogen production (4307.0 kmol h^−1^). This is because the excess amount of water in the hydrogen production process would require an additional amount of glucose that does not stem from cellulose, while insufficient amount of water could be easily added immediately before the hydrogen production process. For the design of the flash distiller, the maximum allowable gas velocity, *V*_a_ [m s^−1^], is calculated by the following equation:2-3-2
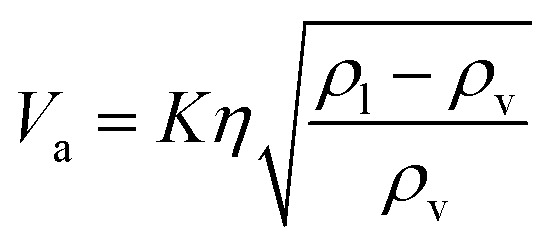


(*K*: rate constant, 0.10 m s^−1^, *η* [−]: design efficiency, 0.85, *ρ*_v_ [kg m^−3^]: vapor density, *ρ*_l_ [kg m^−3^]: liquid density). The drum diameter of the flash drum, *D* [m], is then calculated from *V*_a_:2-3-3
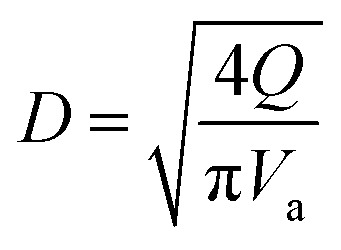


(*Q* [m^3^ s^−1^]: volume flow rate of the vapor). We followed the general design aspect ratio of drums, *H*/*D* = 2.^36^ Optimization of the process conditions was performed to minimize the sum of construction cost and utility cost, by taking the pressure and temperature in the flash distiller as variables.

Glucose thermally decomposes at about 150 °C, which is its melting point,^32^ making it impossible to separate from HMF and levulinic acid, which have boiling points above 150 °C, by a distillation column. Therefore, we employed an extraction column for the separation of glucose. In the extraction column, the design conditions were set so that both the HMF-to-glucose and levulinic acid-to-glucose ratios become below 0.01, to neglect potential side reactions involving HMF and levulinic acid in the subsequent hydrogen production process. MIBK was used as the extractant because it is insoluble in water and due to the molecular properties of MIBK, it interacts well with HMF, which contains polar and non-polar parts, and can extract HMF and levulinic acid well.^35,37,38^ For the design of the extraction column, the continuous phase velocity in flooding, *V*_cf_ [m s^−1^], can be deduced by:2-3-4
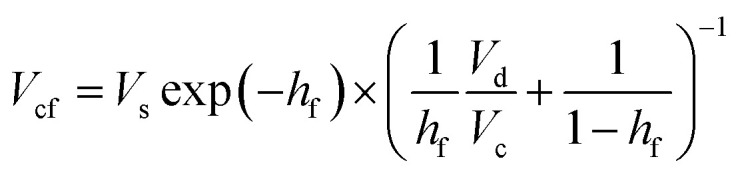


(*V*_s_ [m s^−1^]: slipping phase velocity of droplets, *h*_f_ [−]: holdup of the dispersed layer in flooding, *V*_d_ [m s^−1^]: dispersed phase velocity, *V*_c_ [m s^−1^]: continuous phase velocity). The design velocity of the continuous phase in the column was set to 70% of the flooding velocity, and the column diameter was calculated by:2-3-5
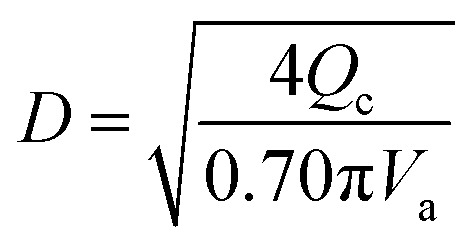


(*Q*_c_ [m^3^ s^−1^]: volume flow rate of the continuous phase). The specific gravity of the residual phase, which is mainly composed of water, is larger than that of the extracted phase, which is mainly composed of MIBK. Therefore, the residual phase was set as the continuous phase and the extracted phase as the dispersed phase. *V*_s_ = 0.035 m s^−1^ and *h*_f_ = 0.4 were used in the calculations. The height of the column was determined by setting the top and bottom trays as 1 m and the distance between the trays as 0.5 m. Water in the oil layer sent from the extraction column is separated by cooling the temperature in the decanter, and the aqueous phase is sent to the hydrogen production process in the same way as the aqueous phase in the extraction column, and the oil layer is sent to the distillation column.

The oil layer sent from the decanter is separated into MIBK and other components by a distillation column. The column is designed to recover more than 99.5% of MIBK. The recovered MIBK is recycled. For the design of the distillation column, the spacing between trays was set as 0.6 m, and the tray efficiency as 0.80. The heights of the top and bottom trays were set as 2 and 4 m, for reflux feed and gas–liquid separation, and for liquid holdup, respectively.^39^ The column diameter of the distillation column is determined based on the allowable vapor mass velocity at which flooding, *etc.* does not occur. In the case of sieve rays, the allowable vapor mass velocity, G^*^ [kg m^−2^ s^−1^], is expressed by the following equation:^39^2-3-6
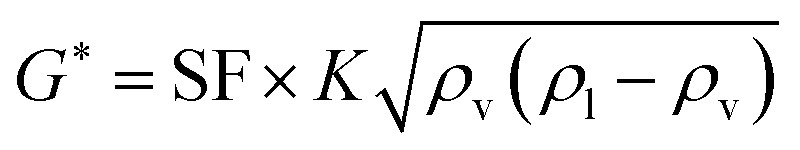


(SF [−]: system correction factor, 0.8, *K*: allowable vapor velocity coefficient, 0.05 m s^−1^). With the calculated G^*^, the column diameter, *D* [m], of the distillation column is determined by:2-3-7
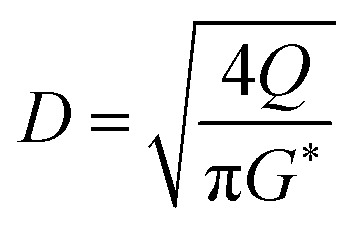


The relationship between the allowable steam velocity and the volume flow rate of the vapor, *Q* [m^3^ s^−1^], determines the conditions that must be met to prevent flooding:2-3-8
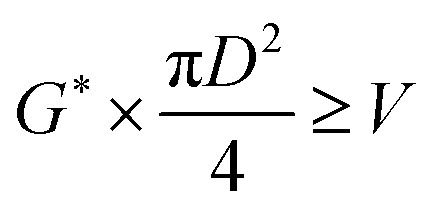


The heat transfer area, *A* [m^2^], of heat exchange is determined by:2-3-9
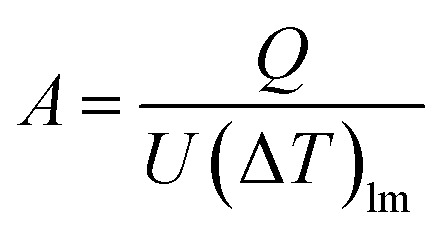


(*Q* [J s^−1^]: heat flow, *U* [W m^−2^ K^−1^]: overall heat transfer coefficient, (Δ*T*)_lm_ [*K*]: logarithmic mean temperature difference). When the temperature of the heat giving fluid changes from *T*_h1_ to *T*_h2_ and the temperature of the heat receiving fluid changes from *T*_c2_ to *T*_c1_, (Δ*T*)_lm_ is determined by:2-3-10
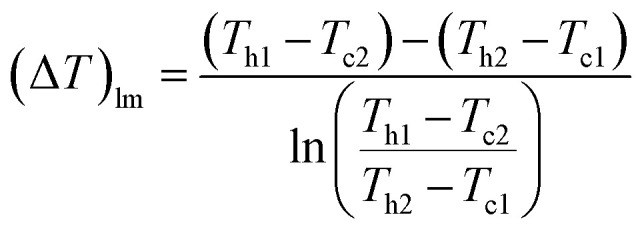


The heat exchanger fluid was assumed to flow in a countercurrent flow because the temperature difference change is generally smaller in a heat exchanger in a countercurrent flow than in a parallel flow. The values of the overall heat transfer coefficient, *U*, were taken from Table 1.^40^ Optimization of the process conditions for the glucose separation process, excluding the flash distiller, was performed to minimize the sum of the extraction column cost, decanter cost, and distillation column cost, by taking the flow rate of MIBK entering the extraction column as a variable.

**Table:** Values of the overall heat transfer coefficient, *U*

Heat giving fluid	Heat receiving fluid	*U* [W m^−2^ K^−1^]
Gas (condensing)	Liquid (vaporizing)	1500
Gas (condensing)	Liquid	1000
Gas (condensing)	Gas	500
Gas	Liquid (vaporizing)	500
Gas	Liquid	200
Gas	Gas	150
Liquid	Liquid (vaporizing)	1000
Liquid	Liquid	300
Liquid	Gas	200
Liquid	Gas (condensing)	1000

### 2.4. Hydrogen production process

In the hydrogen production process, glucose obtained from the glucose separation process is subjected to a supercritical water gasification reaction to obtain hydrogen. The reaction equation of the supercritical water gasification reaction is:2-4-1
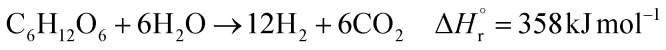
and its reaction rate, denoting *C*_glucose_ [kmol m^−3^] as the glucose concentration, is:^41^

2-4-2 *r* = kC^1.3^_glucose_ [kmol m^−3^ h^−1^]

2-4-3
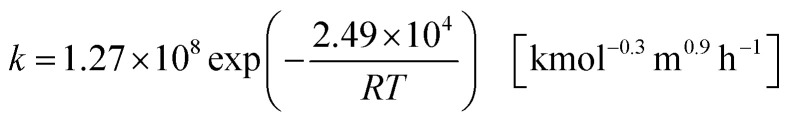


H_2_O, H_2_, and CO_2_ were considered supercritical, glucose and sodium sulfate are dissolved in water, and all other substances were considered as gas phase. For thermal calculations, the temperature- and pressure-dependent molar heat capacity at constant pressure of H_2_O, CO_2_, and H_2_ reported in ref. 42–44 respectively, and of the other chemical species extracted from Aspen HYSYS were employed. The volumetric mass density of H_2_O, CO_2_, and H_2_ also referred to ref. 42–44 respectively.

We employ a tube reactor (so-called plug flow reactor, PFR) for this hydrogen production reaction. As we numerically tested simulating the temperature evolution in the reactor with a condition of the reactor pressure of 26 MPa and the outlet temperature of 650 °C, the maximum temperature change was found to be as small as −17 °C even for the conversion of glucose of 0.99. Therefore, we employed an adiabatic tube reactor. Hastelloy was selected as the reactor material because this reaction is carried out under high temperature and high pressure conditions.

The mass and heat balance equations for the hydrogen production reaction are:2-4-4
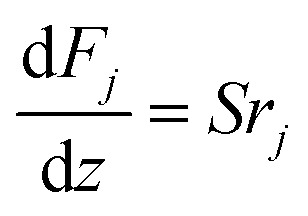
2-4-5
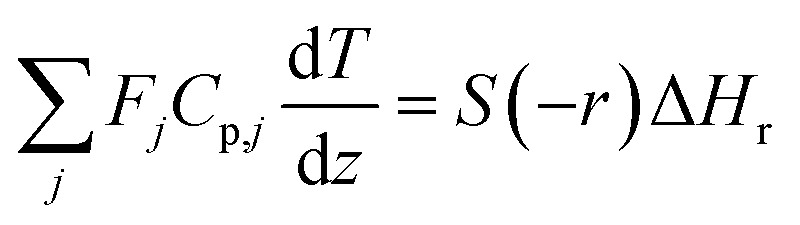


(*F*_*j*_ [kmol h^−1^]: molar flow rate of the component *j*, *z* [m]: position in the tube reactor relative to the inlet, *S* [m^2^]: cross-sectional area of the reactor, *r*_*j*_ [kmol m^−3^ h^−1^]: reaction rate of the component *j*, *C*_p,*j*_ [kJ mol^−1^ K^−1^]: molar heat capacity at constant pressure of the component *j*, *T* [*K*]: temperature in the reactor, Δ*H*_r_ [kJ kmol^−1^]: reaction enthalpy at the temperature *T*). The reaction was numerically simulated by using the fourth-order Runge–Kutta–Gill method for the above simultaneous differential equations. For the design of the reactor, the following assumptions were employed: pressure drop is neglected; the flow in the reactor is an extruded flow; concentration distribution in the reactor is uniform in radial direction; no reaction takes place except for glucose and water; H_2_O, CO_2_, and H_2_ are supercritical; glucose and sodium sulfate are dissolved in water; HMF, levulinic acid, formic acid, and MIBK are in the gas phase; formic acid is immediately decomposed at the reactor entrance according to the following equation:2-4-6
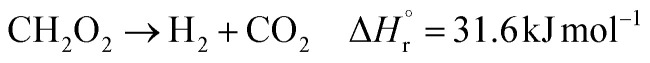


By referring ref. 41, the design conditions were set as follows: the concentration of supplied glucose solution of 5 wt%, the reactor pressure between 23 and 30 MPa, the reactor temperature between 650 and 700 °C. The length-to-diameter (*L*/*D*) ratio of the reactor was designed to be 3, since an *L*/*D* ratio of 2 to 4 is commonly employed. Optimization of the process conditions was performed to minimize the sum of the construction cost for the reactor, heat exchanger, and furnace, the utility cost, and the pressurization cost, by taking the reactor pressure and the outlet temperature as variables. It was assumed that the furnace has a combustion efficiency of 50% and excess air of 50%, and that hexane is purchased as fuel to burn hexane at 900 °C.

### 2.5. Hydrogen separation process

**Fig. 4:**
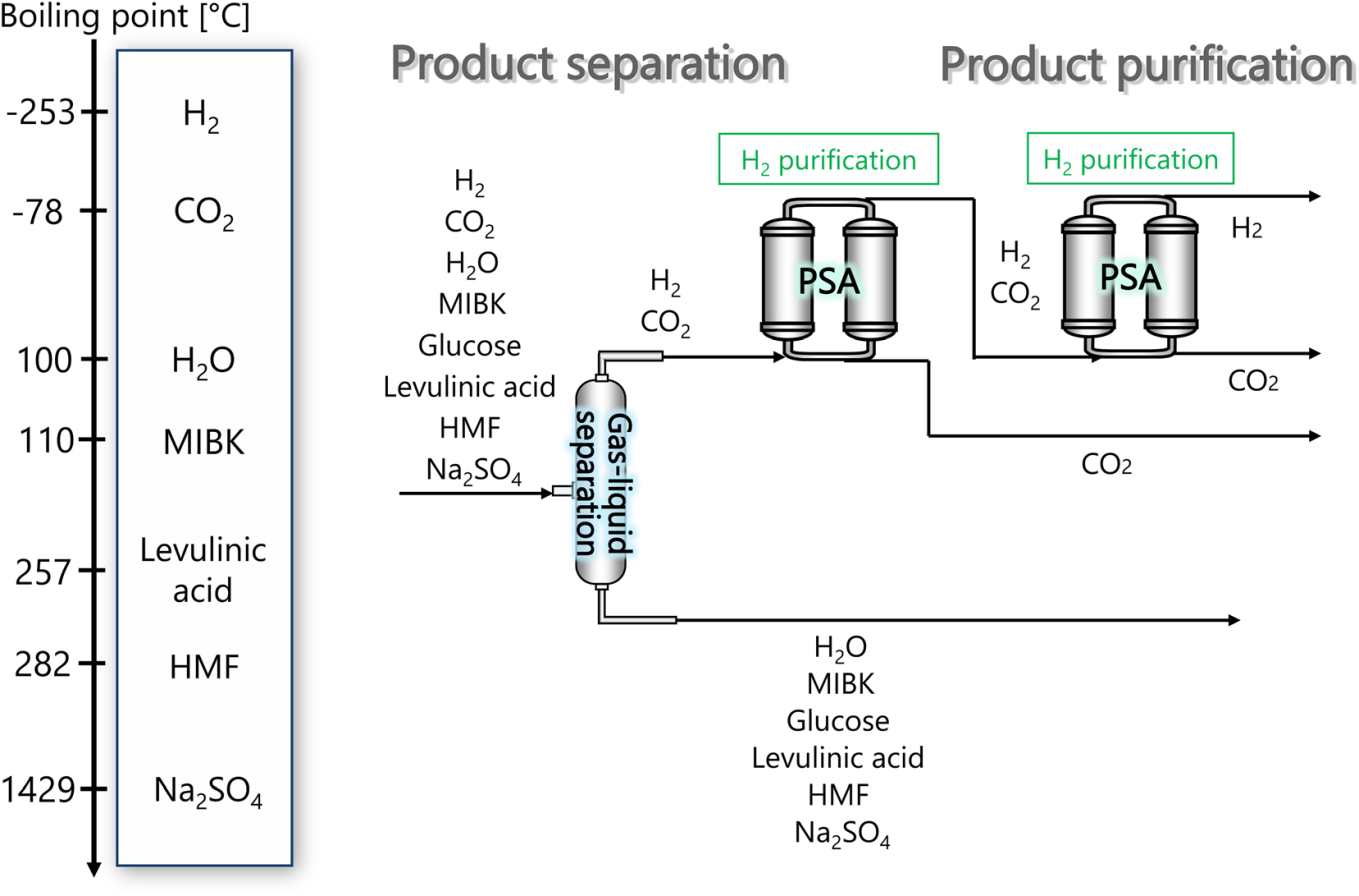
Flow diagram of the hydrogen separation process, along with the boiling points of the involved components. MIBK is methyl isobutyl ketone, HMF is hydroxymethylfurfural, and PSA is pressure swing adsorption.

In the hydrogen separation process, the components created by the hydrogen production process are separated to obtain hydrogen with a purity of 99.99 vol% or higher as a product. Fig. 4 presents the flow diagram of the hydrogen separation process and the boiling points of the involved components. First, H_2_ and CO_2_ in the gas phase are separated from other components in the liquid phase in a gas–liquid separator. The separated gas components then flow into the pressure swing adsorption (PSA) process, where H_2_ and CO_2_ are separated, and H_2_ is purified and made into products.

The pressure of the flow out of the previous hydrogen production process is 260 bar. However, a large amount of H_2_ is dissolved in the liquid phase if the gas–liquid separation is performed at 260 bar. Therefore, the pressure was reduced to 30 bar before the gas–liquid separation, because the operating pressure of the adsorption column in the subsequent PSA process is between 10 and 30 bar. Since the components other than H_2_ and CO_2_ become gaseous at 650 °C, the gas–liquid separation was performed after cooling down to 25 °C. The gas–liquid separation process was designed in the same manner as for the flash distiller in Section 2.3.

For the separation of H_2_ and CO_2_, PSA was used. PSA is an operation that performs adsorption under high pressure and desorption under reduced pressure to separate the bulk of the gas mixture. Continuous adsorption operation is possible by switching the roles of the adsorption and desorption columns. The design conditions of PSA were set as follows: the resulting hydrogen purity is larger than 99.99 vol%; the operating pressure of adsorption column is between 10 and 30 bar; the pressure of desorption column is 0.2 bar; activated carbon is used as adsorbent; temperature dependence of the adsorption isotherm is taken into account; adsorbent replacement period is 1 year; no recycle of hydrogen is operated during desorption; adsorption of the components other than H_2_ and CO_2_ is negligible because of their small amount; the two-column system is employed; the pressure drop is smaller than 0.1 bar; the operating temperature is room temperature; the switchover time is 300 s; the desorption time is not taken into account.

**Fig. 5:**
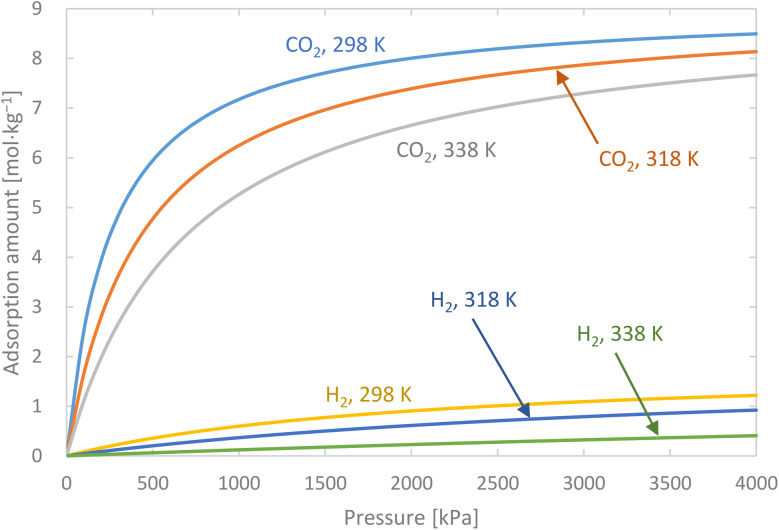
Adsorption isotherms of CO_2_ and H_2_ for the activated carbon.

Activated carbon was employed as the adsorbent in the PSA process. The physical property values and kinetic parameters, such as the Langmuir constant and saturation adsorption amount of CO_2_ and H_2_, of the activated carbon reported in ref. 45 were used for our calculations. The adsorption isotherms of CO_2_ and H_2_ for the activated carbon are presented in Fig. 5. It is observed in the isotherms that a small amount of H_2_ is adsorbed along with CO_2_ under high pressure. Therefore, assuming adsorption of two components, CO_2_ and H_2_, the following Markham–Benton equation, which is an extension of the Langmuir equation to a multi-component system, was employed as the adsorption isotherm equation.2-4-7
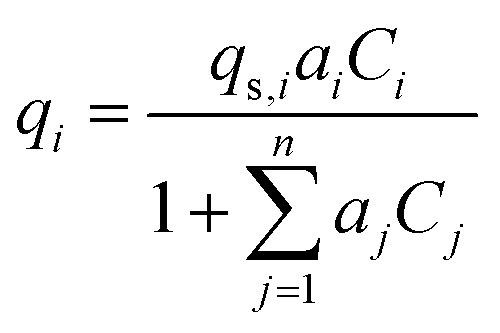


(*q*_*i*_ [mol kg^−1^]: adsorption amount of the component *i* per mass of the adsorbent, *q*_s,*i*_ [mol kg^−1^]: saturation adsorption amount of the component *i* per mass of the adsorbent, *a*_*i*_ [m^3^ mol^−1^]: concentration-based Langmuir constant, *C*_*i*_ [mol m^−3^]: concentration of the adsorbate *i*) the pressure drop was estimated by the following Kozeny–Carman equation, with Kozeny constant *k* = 5.2-4-8
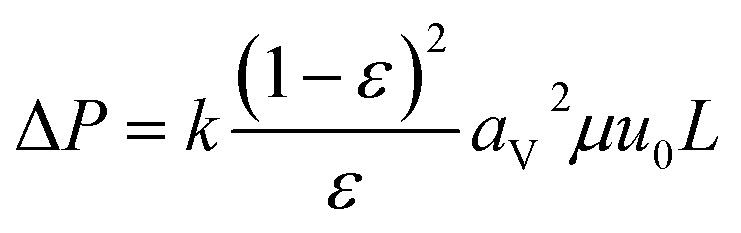


(Δ*P* [Pa]: pressure drop, *k* [−]: Kozeny constant, *ε* [−]: porosity, *a*_v_ [m^2^ m^−3^]: surface area of the particles in the unit volume of packed column, *μ* [kg m^−1^ s^−1^]: viscosity of the fluid, *u*_0_ [m s^−1^]: superficial velocity, *L* [m]: column length) the switching of columns were operated at the breakthrough time to increase the purity of H_2_.

The mass balance and adsorption rate equations for the design of the PSA process are:2-4-9
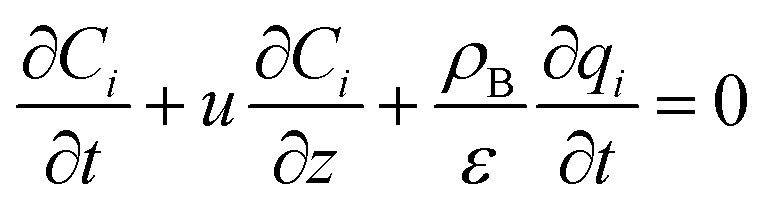
2-4-10
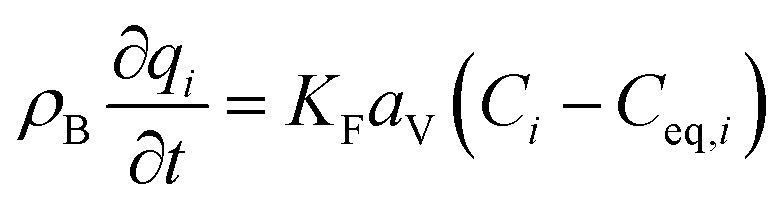


(*u* [m s^−1^]: line velocity, *ρ*_B_ [kg m^−3^]: packing density of the activated carbon, *K*_F_*a*_v_ [s^−1^]: overall mass transfer capacity coefficient, *C*_eq,*i*_ [mol m^−3^]: equilibrium concentration of the adsorbate *i*), respectively. It was assumed that: the PSA process is in an isothermal system; the gas behaves as ideal gas; the gas follows an extruded flow in the column; the superficial velocity of the gas is spatiotemporally constant; the adsorbate concentration at the inlet is temporally constant; the condition of the constant concentration profile holds; the adsorbate concentration is uniform in the radial direction in the column; the adsorbent is homogeneous spherical particles; the species are in equilibrium at the outer surface of the adsorbent particles; the increase and decrease of pressure are promptly completed; the desorption duration is shorter than the adsorption duration. Since the overall mass transfer is governed by the diffusion resistance between the adsorbent particles and the fluid and that inside the particles, the overall mass transfer capacity coefficient, *K*_F_*a*_v_, was estimated by:2-4-11
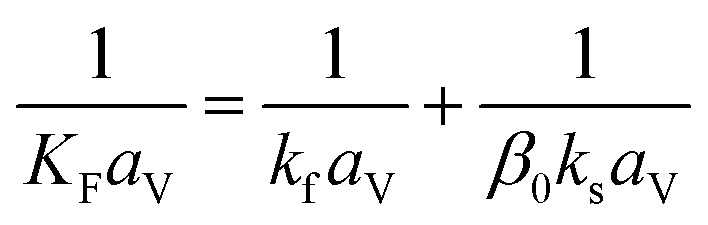


(*k*_f_ [m s^−1^]: film mass transfer coefficient between the particle and fluid, *β*_0_ [m^3^ kg^−1^]: adsorption coefficient, *k*_s_ [kg m^−2^ s^−1^]: film mass transfer coefficient for inner-particle diffusion). The film mass transfer coefficient between the particle and fluid, *k*_f_, was deduced from the following Carberry equation:2-4-12
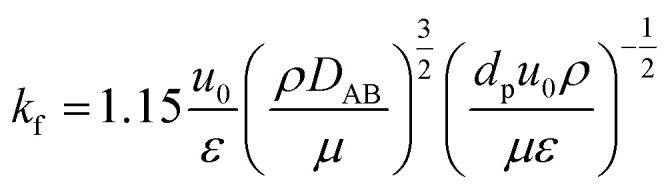


(*ρ* [kg m^−3^]: volumetric mass density of the fluid, *D*_AB_ [m^2^ s^−1^]: molecular diffusion coefficient, *d*_p_ [m]: diameter of the activated carbon particles). The film mass transfer coefficient for inner-particle diffusion, *k*_s_, was deduced from the following equation proposed by Glueckauf:2-4-13
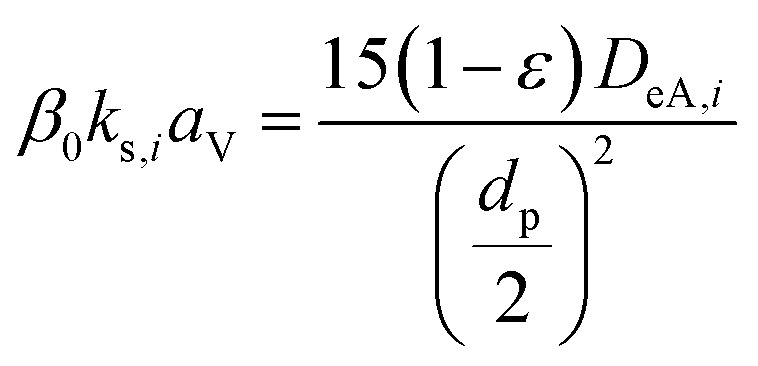


(*D*_eA,*i*_ [m^2^ s^−1^]: effective inner-particle diffusion coefficient of the component *i*). Let us present the method for estimating the physical quantities needed to calculate *k*_f_ and *k*_s,*i*_. The following Chapman–Enskog equation was used to estimate the viscosity, *μ*, of pure substances:2-4-14
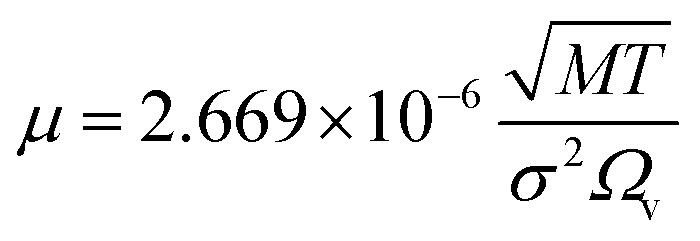


(*M* [g mol^−1^]: molecular mass, *σ* [Å]: molecular collision diameter). The reduced collision integral, *Ω*_v_ [−], was determined by:2-4-15
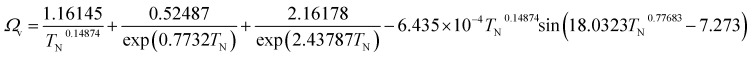
2-4-16
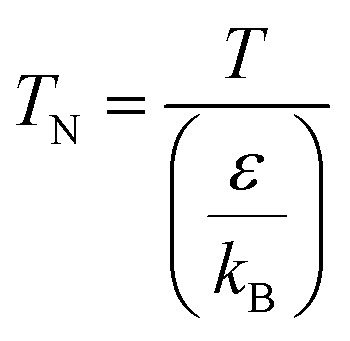


For the Chapman–Enskog equation, *M* is 44.01 and 2.016 g mol^−1^, *σ* is 3.996 and 2.915 Å, *ε*/*k*_B_ (the Lennard–Jones parameter) is 190 and 38 K for CO_2_ and H_2_, respectively.^46^ The viscosity of the gas mixture was calculated using the following Sutherland equation from the viscosity of each component determined by the Chapman–Enskog equation:2-4-17
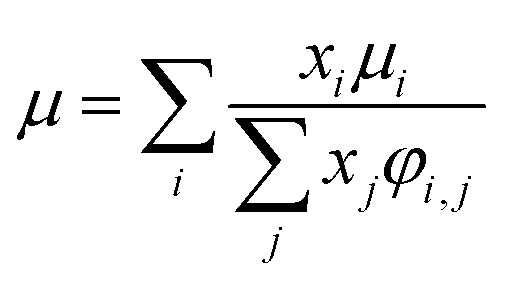


(*x*_*i*_ [−]: molar fraction of the component *i*, *φ*_*i*,*j*_ [−]: connection coefficient between the components *i* and *j*). *φ*_*i*,*j*_ was determined by the following Wike equation:2-4-18
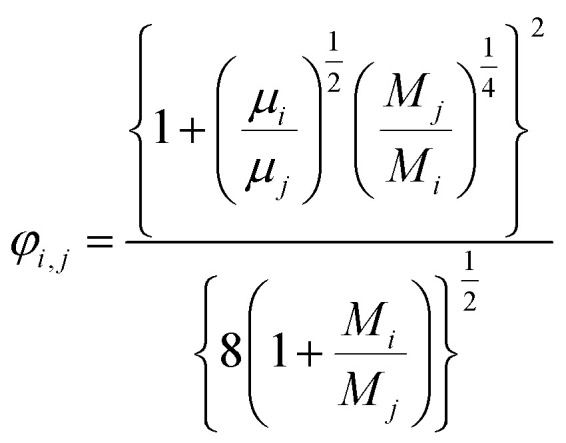


(*M*_*i*_ [g mol^−1^]: molecular mass of the component *i*). The volumetric mass density of the gas mixture was calculated from the ideal gas equation of state as an ideal gas:2-4-19
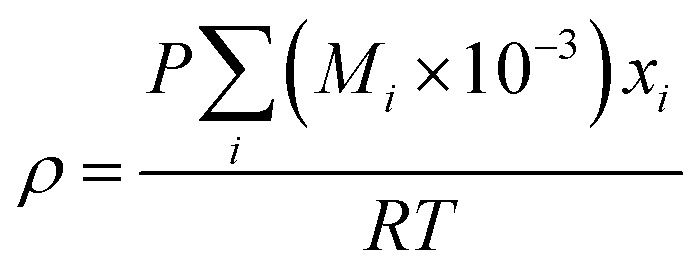


The molecular diffusion coefficient of the gas mixture, *D*_AB_ [m^2^ s^−1^], was deduced from the Chapman–Enskog theory equation:2-4-20
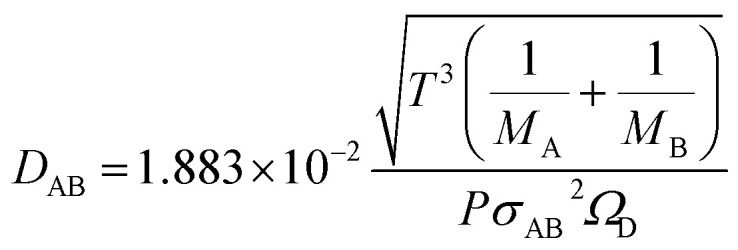
2-4-21
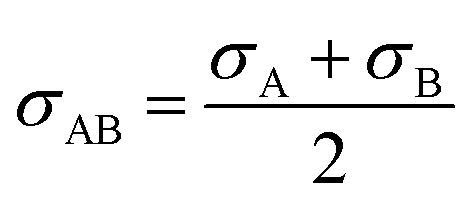
2-4-22
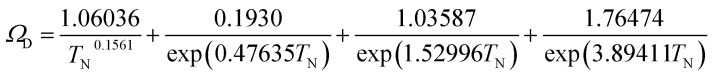
2-4-23
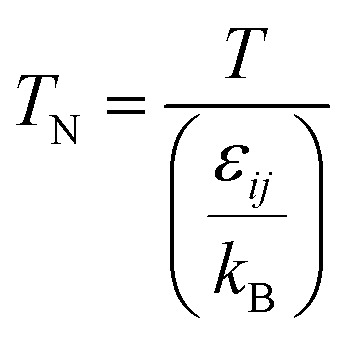
2-4-24
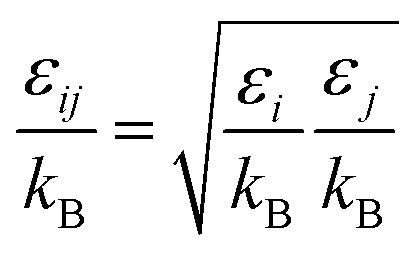


(*P* [Pa]: pressure, *Ω*_D_ [−]: reduced collision integral for diffusion). The effective inner-particle diffusion coefficient, *D*_eA,*i*_, was estimated from the random pore model, which takes into account macro- and micro-pores, using the following equation:2-4-25
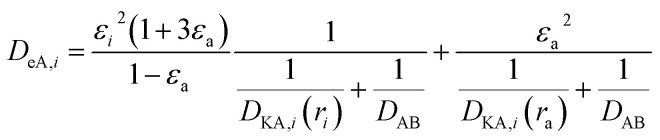


(*ε*_*i*_ [−]: microporosity, *ε*_a_ [−]: macroporosity, *r*_*i*_ [m]: radius of the micropores, *r*_a_ [m]: radius of the macropores). The Knudsen diffusion coefficients for micro- and macropores, *D*_KA,*i*_ [m^2^ s^−1^], were obtained from the following equation:2-4-26
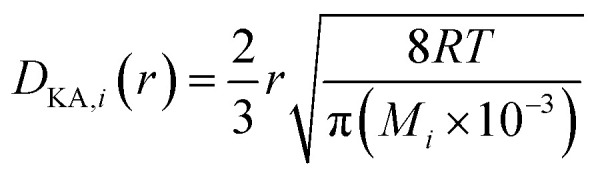


(*R* [J mol^−1^ K^−1^]: gas constant, *r* [m]: radius of the pores). Optimization of the process conditions of PSA was performed to minimize the sum of hydrogen loss cost, construction cost, and adsorbent cost, by taking the pressures of the first and second adsorption columns as variables.

### 2.6. Levulinic acid separation process

**Fig. 6:**
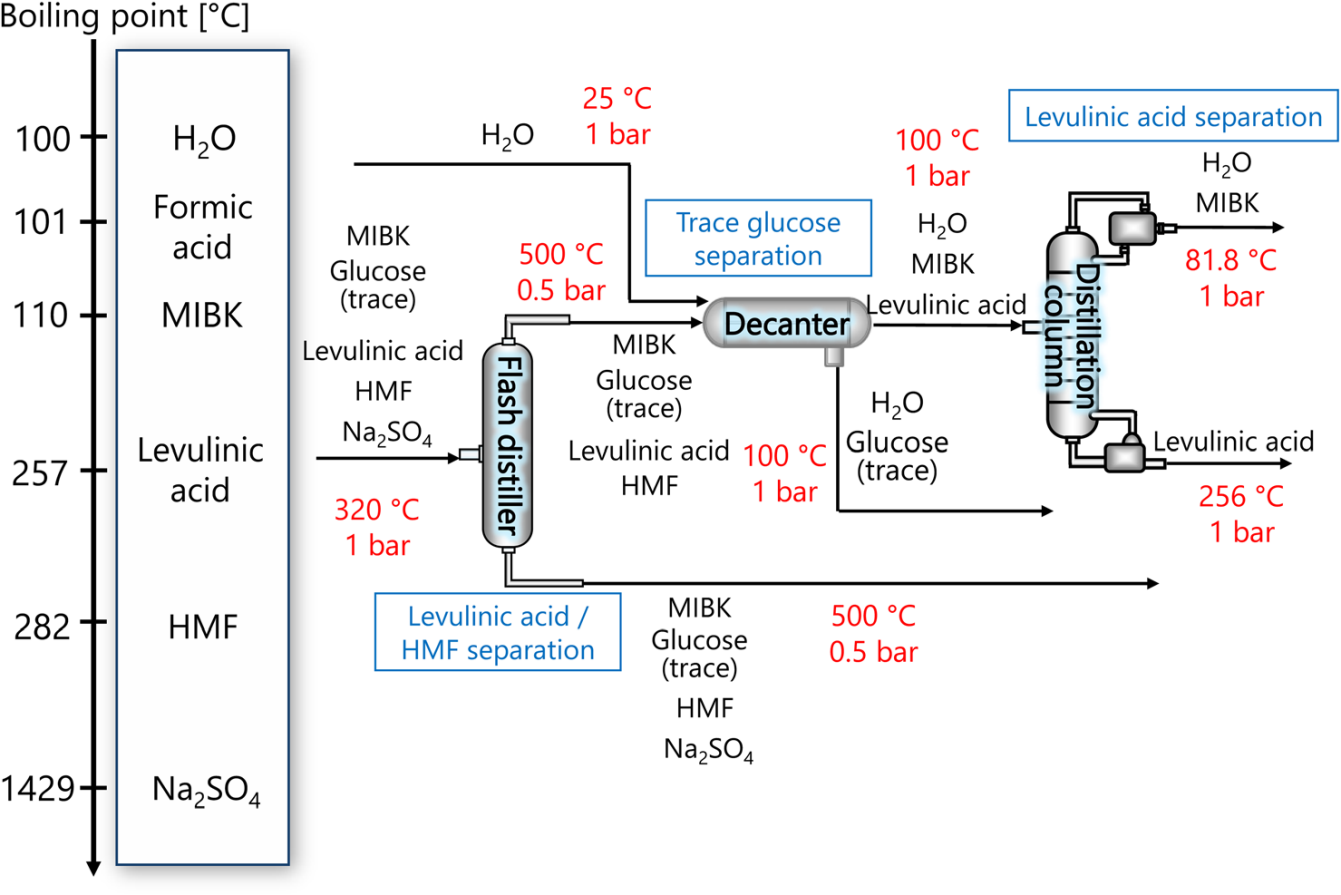
Flow diagram of the levulinic acid hydrogen separation process, along with the boiling points of the involved components. Note that glucose has no boiling point because it thermally decomposes above its melting point, 150 °C.^32^ MIBK is methyl isobutyl ketone and HMF is hydroxymethylfurfural.

The purpose of the levulinic acid separation process is to obtain levulinic acid with a purity of 99% or higher as a product from the components obtained from the glucose separation process. Fig. 6 presents the flow diagram of the levulinic acid separation process and the boiling points of the involved components. Note that glucose has no boiling point because it thermally decomposes above its melting point, 150 °C.^32^ First, HMF and levulinic acid are separated in a flash distillation unit, and a decanter is installed to separate the trace amount of glucose contained in the gas component. The oil phase component is then purified in a distillation column to 99% purity levulinic acid.

The flash distiller was designed to achieve a levulinic acid yield of higher than 95%. Simple succession of the outlet pressure and temperature of the hydrogen separation process, 1 bar and 320 °C, respectively, as the inlet condition of the flash distiller failed in sufficient separation, the pressure in the flash distillation unit was reduced to 0.5 bar and the temperature was increased to 500 °C. The flash distiller in the levulinic acid separation process was designed in the same manner as for the flash distiller in the glucose separation process, as described in Section 2.3.

In the decanter, in addition to the inlet component, the addition of H_2_O is intended to separate the glucose, which is present in trace amounts, to increase the purity of the levulinic acid by extraction. The oil layer sent from the decanter is separated in a distillation column into levulinic acid and other components. The distillation column was designed to achieve a purity of 99% or higher for the levulinic acid. The distillation column in this levulinic acid separation process was designed in the same manner as for the distillation column in the glucose separation process described in Section 2.3.

### 2.7. Cost calculations

For the cost calculations, the bare-module-cost-estimation scheme^47^ was employed. Table 2 lists the symbols of parameters used in this subsection. The following equations were used to calculate the plant construction cost, *C*_BM_ [US$]:

2-7-1 log_10_ *C*_p_ = *K*_1_ + *K*_2_ log_10_ *A* + *K*_3_ (log_10_ *A*)^2^

2-7-2 log_10_ *F*_p_ = *C*_1_ + *C*_2_ log_10_ *P*_g_ + *C*_3_ (log_10_ *P*_g_)^2^

2-7-3 *C*^0^_BM_ = (*B*_1_ + *B*_2_*F*_M_*F*_p_)*C*_p_

2-7-4
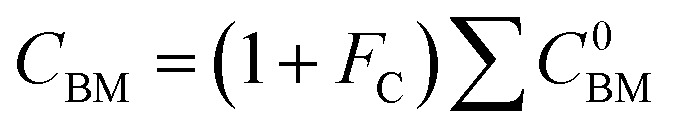
2-7-5
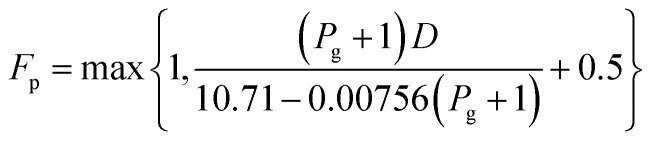


**Table:** List of the symbols of parameters, their definitions, and units used in Section 2.7

*A*	Characteristic size of equipment	(Table 3)
*B*_1_	Coefficient for the part independent of pressure and equipment material	[−]
*B*_2_	Coefficient for the part dependent on pressure and equipment material	[−]
*b*	Blade width	[m]
*C*_BM_	Plant construction cost	[US$]
*C* ^0^_BM_	Cost of equipment installation including direct and indirect costs	[US$]
*C*_*i*_	Constants depending on equipment type	[−]
*C*_p_	Equipment purchase cost for atmospheric pressure operation and carbon steel materials	[US$]
*D*	Tube or tank diameter	[m]
*d*	Blade diameter	[m]
*F*_C_	Cost factor accounting for losses and spare parts costs	[−]
*F*_M_	Correction term for equipment material	[−]
*F*_p_	Correction term for operating pressure	[−]
*H*	Liquid depth	[m]
*K*_*i*_	Constants depending on equipment type	[−]
*N*_p_	Number of motive forces	[−]
*n*	Rotation velocity	[s^−1^]
*P*	Power required for stirring	[W]
*P*_g_	Operating pressure of equipment	[bar]
Re	Impeller Reynolds number	[−]
*ρ*	Liquid density	[kg m^−3^]

(*C*_p_ [US$]: equipment purchase cost for atmospheric pressure operation and carbon steel materials, *K*_*i*_ [−]: constants depending on equipment type, *A* [unit follows Table 3]: characteristic size of equipment, *F*_p_ [−]: correction term for operating pressure, *C*_*i*_ [−]: constants depending on equipment type, *P*_g_ [bar]: operating pressure of equipment, *C*^0^_BM_ [US$]: cost of equipment installation including direct and indirect costs, *B*_1_ [−]: coefficient for the part independent of pressure and equipment material, *B*_2_ [−]: coefficient for the part dependent on pressure and equipment material, *F*_M_ [−]: correction term for equipment material, *F*_C_ [−]: cost factor accounting for losses and spare parts costs, *D* [m]: tube diameter). Table 3 lists the coefficients used in the equations above. The electrically driven stirring costs were calculated from the following equations:

2-7-6 *P* = *ρN*_P_*n*^3^*d*^5^

2-7-7
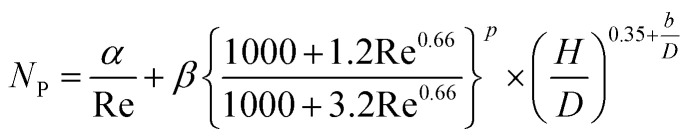
2-7-8
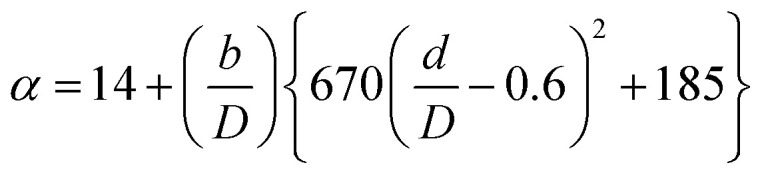
2-7-9
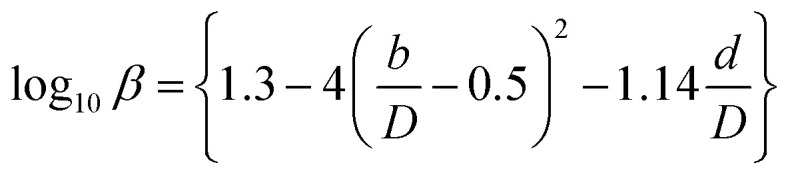
2-7-10
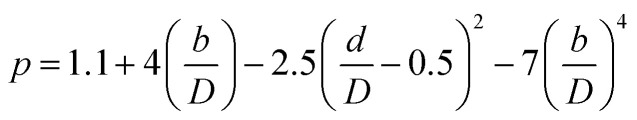


**Table:** Coefficients for the calculation of plant construction cost. CSTR is continuous stirred-tank reactor

	*K*_1_	*K*_2_	*K*_3_	*B*_1_	*B*_2_	*A*	*F*_M_	*F*_C_
CSTR	4.1052	0.5320	−0.0005	2.25	1.82	Volume [m^3^]	7.1	0.18
Tube reactor	3.5565	0.3776	0.0905	1.49	1.52	Volume [m^3^]	7.1	0.18
Column	3.4974	0.4485	0.1074	2.25	1.82	Volume [m^3^]	4.8	0.18
Reboiler	4.4646	−0.5277	0.3955	1.63	1.66	Area [m^2^]	1.7	0.18
Condenser	4.8306	−0.8509	0.3187	1.63	1.66	Area [m^2^]	1.7	0.18
Heat exchanger	3.3444	0.2745	−0.0472	1.74	1.55	Area [m^2^]	1.0	0.18
Pump	3.3892	0.0536	0.1538	1.89	1.35	Power [kW]	4.8	0.18
Decanter	3.4974	0.4485	0.1074	2.25	1.82	Volume [m^3^]	3.9	0.18
Furnace	7.3488	−1.1666	0.2028	1.00	1.00	Heat [kW]	1.0	0.18

(*P* [W]: power required for stirring, *ρ* [kg m^−3^]: liquid density, *N*_p_ [−]: number of motive forces, *n* [s^−1^]: rotation velocity, *d* [m]: blade diameter, Re [−]: impeller Reynolds number, *H* [m]: liquid depth, *D* [m]: tank diameter, *b* [m]: blade width). *d*/*D* = 0.5 and *b*/*D* = 0.1 were assumed as two-blade paddles.

Prices of the raw materials and products are listed in Table 4.^48–52^ Cellulose was assumed to be *Cenchrus purpureus*,^53^ which costs $0.016 kg^−1^ as raw material, and $0.0977 kg^−1^ including the cost of pretreatment such as pulverization.^54^ The prices of utilities are shown in Table 5.^39,55^ The combustion heat of hexane was read from simulation results of an Aspen HYSYS furnace (50% combustion efficiency, 50% excess air).

**Table:** Prices of the raw materials and products. MIBK is methyl isobutyl ketone

Raw material	Price
Cellulose	$0.0977 kg^−1^
Sulfuric acid	$0.0477 kg^−1^
Water	$0.00022 kg^−1^
NaOH	$298 ton^−1^
MIBK	$1510 ton^−1^
Activated carbon	$1.95 kg^−1^
Hydrogen (revenue)	$0.769 Nm^−3^
Levulinic acid (revenue)	$10 kg^−1^

**Table:** Prices of the utilities. The latent heat of evaporation corresponds to the low-pressure steam, the heat of combustion to the fuel hexane, and *C*_p_Δ*T* to the cooling water

Utility	Temperature	Price	Latent heat of evaporation, heat of combustion, *C*_p_Δ*T*
High-P steam	500 °C	$31 GJ^−1^	
Low-P steam	160 °C	$15 ton^−1^	2081.3 kJ kg^−1^
Cooling water	30 °C supply/40 °C return	$0.013 ton^−1^	41.78 kJ kg^−1^
Propylene coolant	5 °C	$4.43 GJ^−1^	
Electric power		$0.1 kW h^−1^	
Fuel hexane		$0.23 kg^−1^	23 578 kJ kg^−1^

## 3. Results and discussion

### 3.1. Glucose production process

**Fig. 7:**
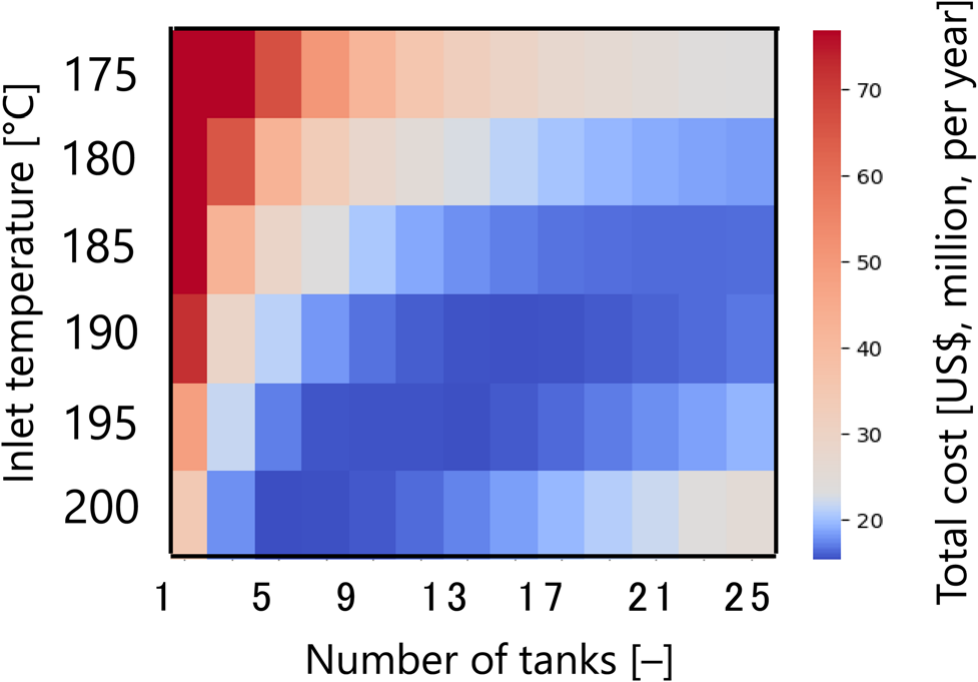
Dependence of the total cost on the inlet temperature and the number of tanks in the glucose production process, with a fixed residence time of 13 s and sulfuric acid concentration of 0.3 mol L^−1^. The total cost refers to the sum of construction cost, utility cost, and raw material cost for the reactor and heat exchanger.

First, Fig. 7 presents the calculation result of the dependence of the total cost for the target hydrogen production (41 million Nm^3^ per year, accompanied with conditions described in Section 2.1) on the inlet temperature and the number of tanks in the glucose production process, with a fixed residence time of 13 s and sulfuric acid concentration of 0.3 mol L^−1^. The total cost refers to the sum of construction cost, utility cost, and raw material cost for the reactor and heat exchanger, as described in Section 2.2. In the region where both the inlet temperature and the number of tanks are small, the glucose production reaction does not proceed, and therefore the raw material cost becomes large. On the other hand, in the region where both the inlet temperature and the number of tanks are large, the glucose selectivity decreases due to excessive side reactions, also resulting in a large raw material cost. In other regions, higher inlet temperatures increase the utility cost, but the reaction rate increases and the number of tanks required decreases, resulting in lower construction costs. Based on the trends described above, a minimum point of total cost emerges, and the optimal inlet temperature and number of tanks were found to be 200 °C and five, respectively.

**Fig. 8:**
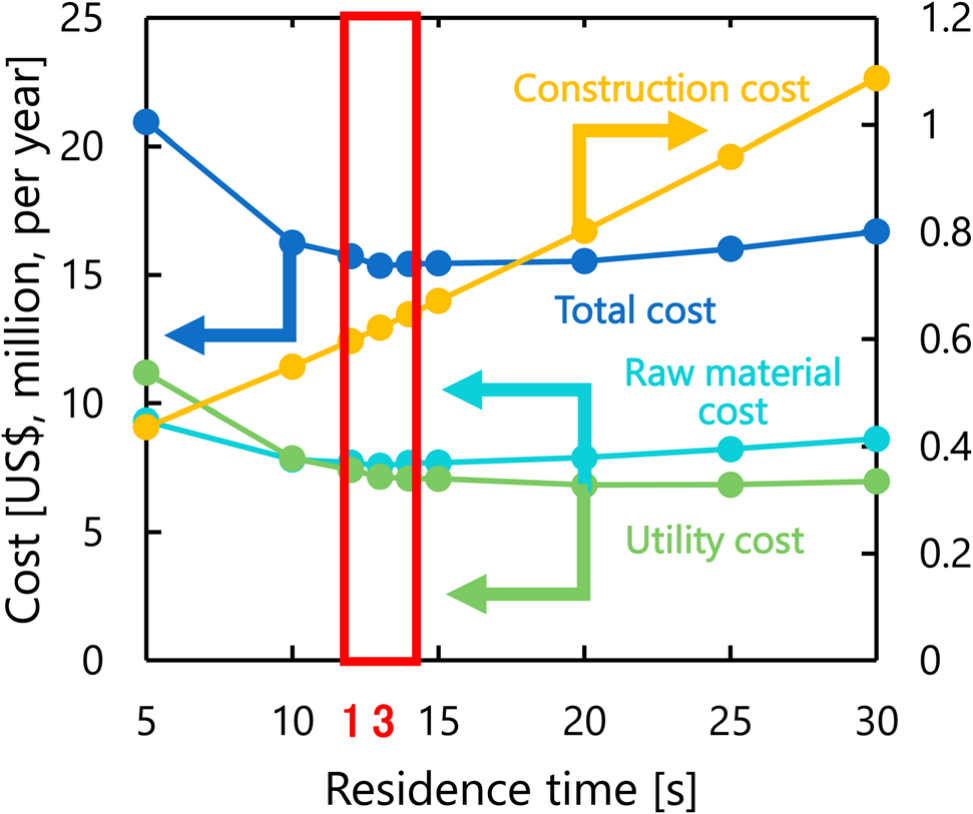
Dependence of the construction cost, utility cost, raw material cost, and total cost on the residence time with a fixed inlet temperature of 200 °C, number of tanks of five, and sulfuric acid concentration of 0.3 mol L^−1^.

Fig. 8 presents the dependence of the construction cost, utility cost, raw material cost, and total cost on the residence time with a fixed inlet temperature of 200 °C, number of tanks of five, and sulfuric acid concentration of 0.3 mol L^−1^. As the residence time decreases, the reaction rate decreases, and therefore the raw material cost and utility cost required for heating and cooling the materials increases. Whilst, as the residence time increases, the glucose selectivity decreases, and consequently the raw material cost and utility cost increase slightly and the construction cost significantly increases due to the larger volume of the reactor. As the result of the trends above, the optimal residence time was determined to be 13 s.

**Fig. 9:**
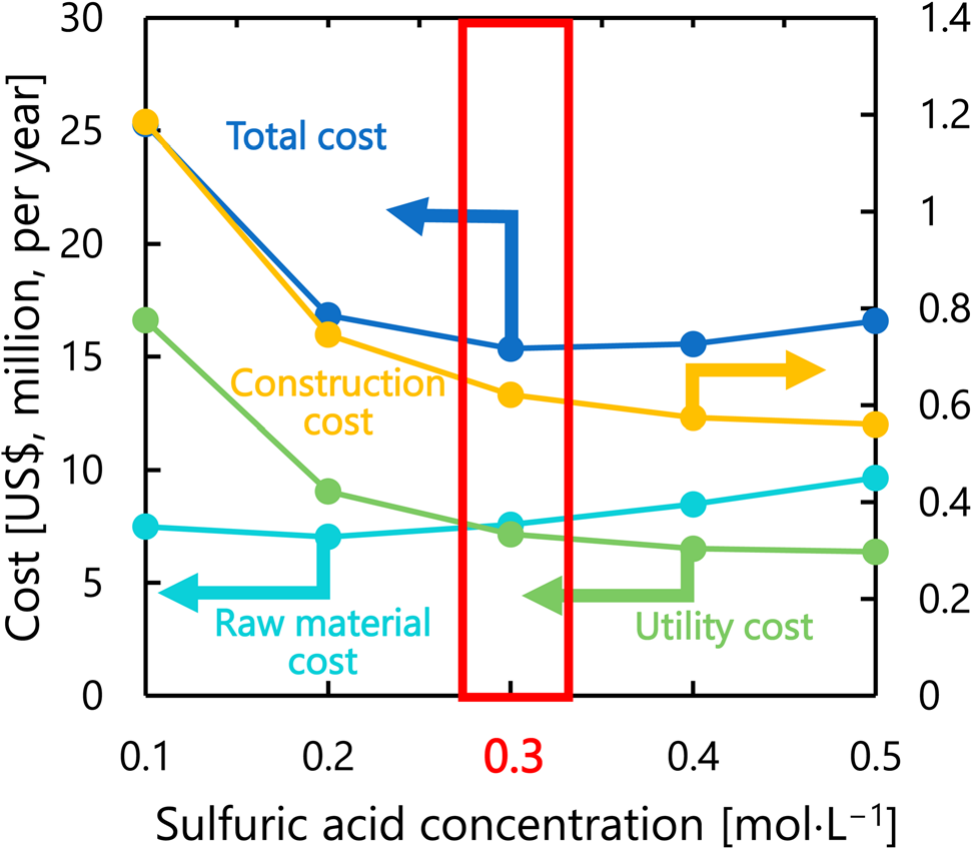
Dependence of the construction cost, utility cost, raw material cost, and total cost on the sulfuric acid concentration with a fixed inlet temperature of 200 °C, number of tanks of five, and residence time of 13 s.

Fig. 9 presents the dependence of the construction cost, utility cost, raw material cost, and total cost on the sulfuric acid concentration with a fixed inlet temperature of 200 °C, number of tanks of five, and residence time of 13 s. As the sulfuric acid concentration increases, the cost of raw materials (sulfuric acid) increases, but the reaction rate increases and the amount of cellulose required decreases, resulting in lower utility cost and construction cost. The optimal sulfuric acid concentration was found to be 0.3 mol L^−1^.

**Fig. 10:**
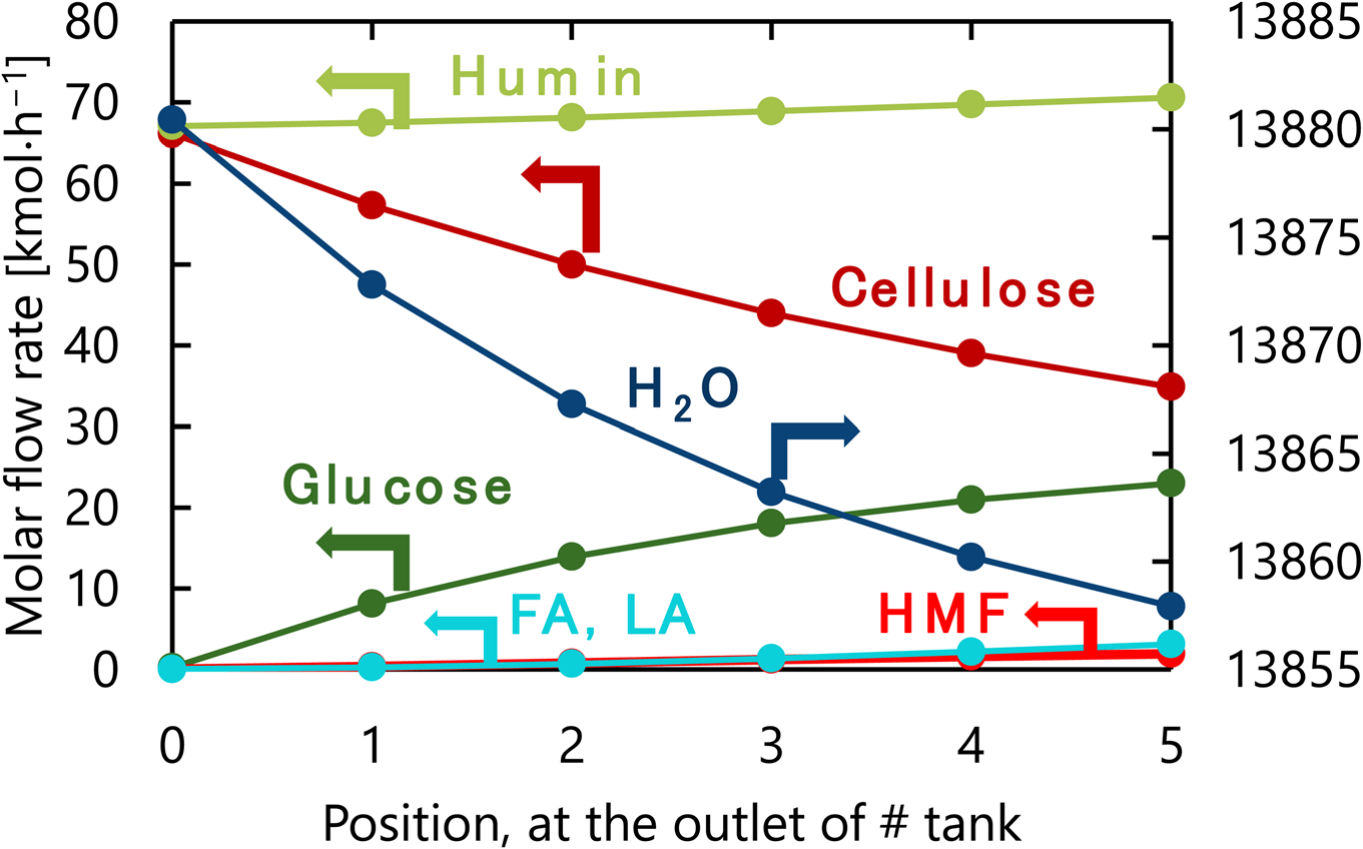
Molar flow rate of each component at the inlet of the first tank and the outlet of each tank of the glucose production process in its optimized condition. FA, LA, and HMF stand for formic acid, levulinic acid, and hydroxymethylfurfural, respectively.

The design of the glucose production reactor resulting from the abovementioned process optimization based on the total cost for the target hydrogen production amount is summarized in Table 6. Fig. 10 presents the molar flow rate of each component at the inlet of the first tank and the outlet of each tank of the glucose production process in its optimized condition. The glucose selectivity was 0.73 as the glucose degradation reaction proceeded. It can also be observed that a large amount of water is flowing through the reactor because of the 4-wt% cellulose solution supplied to the reaction.

**Table:** Summary of the optimal design of the glucose production reactor. CSTR stands for continuous stirred-tank reactor

Type of reactor	CSTR
Reactor height	1.79 m
Reactor diameter	0.90 m
Number of reactor tanks	5
Reactor pressure	16.0 bar
Inlet temperature	200 °C
Outlet temperature	198 °C
Residence time	13 s
Sulfuric acid concentration	0.30 mol L^−1^
Glucose selectivity	0.73

### 3.2. Glucose separation process

**Fig. 11:**
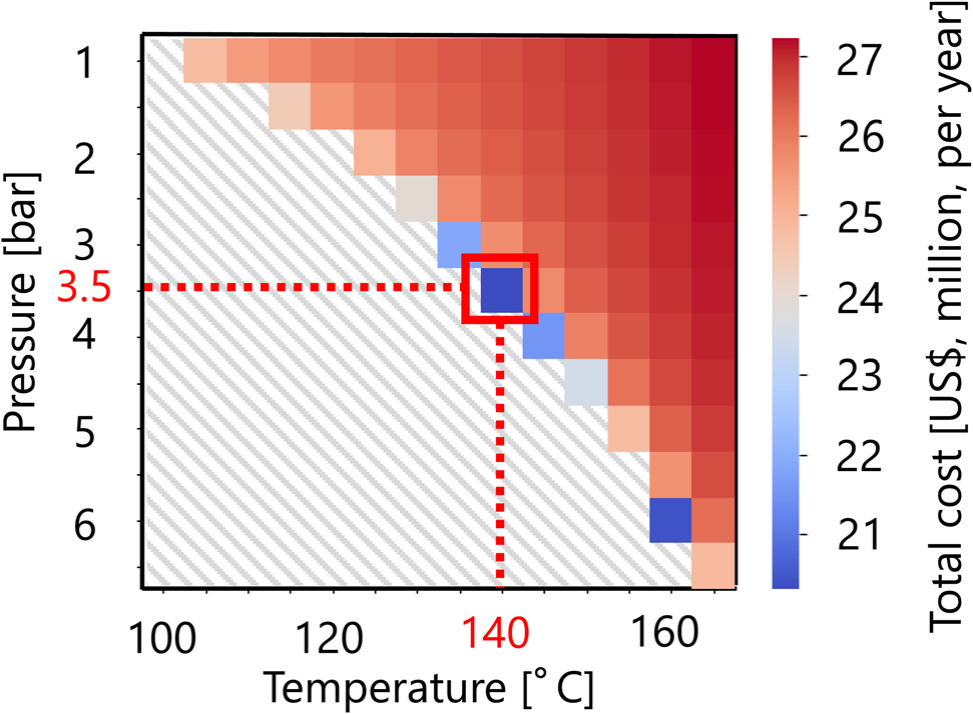
Dependence of the total cost on the pressure and temperature in the flash distiller. The total cost refers to the sum of construction cost and utility cost.

For the flash distiller in the glucose separation process, Fig. 11 presents the calculation result of the dependence of the total cost on the pressure and temperature in the flash distiller. The total cost in this part refers to the sum of construction cost and utility cost, as described in Section 2.3. The smaller the pressure difference between the inlet pressure and the pressure in the flash distiller, the lower the separation cost. On the other hand, the smaller the temperature difference between the inlet temperature and the temperature in the flash distiller, the lower the separation cost. As the result of these competing trends, a minimum point of total cost emerges, and the optimum point was reached at 3.5 bar for the pressure and 140 °C for the temperature in the flash distiller. The design of the flash distiller in the glucose separation process resulting from the abovementioned process optimization based on the total cost for the target hydrogen production amount is summarized in Table 7.

**Table:** Summary of the optimal design of the flash distiller in the glucose separation process

Distiller pressure	3.5 bar
Distiller temperature	140 °C
Column height	8.25 m
Column diameter	4.12 m

**Fig. 12:**
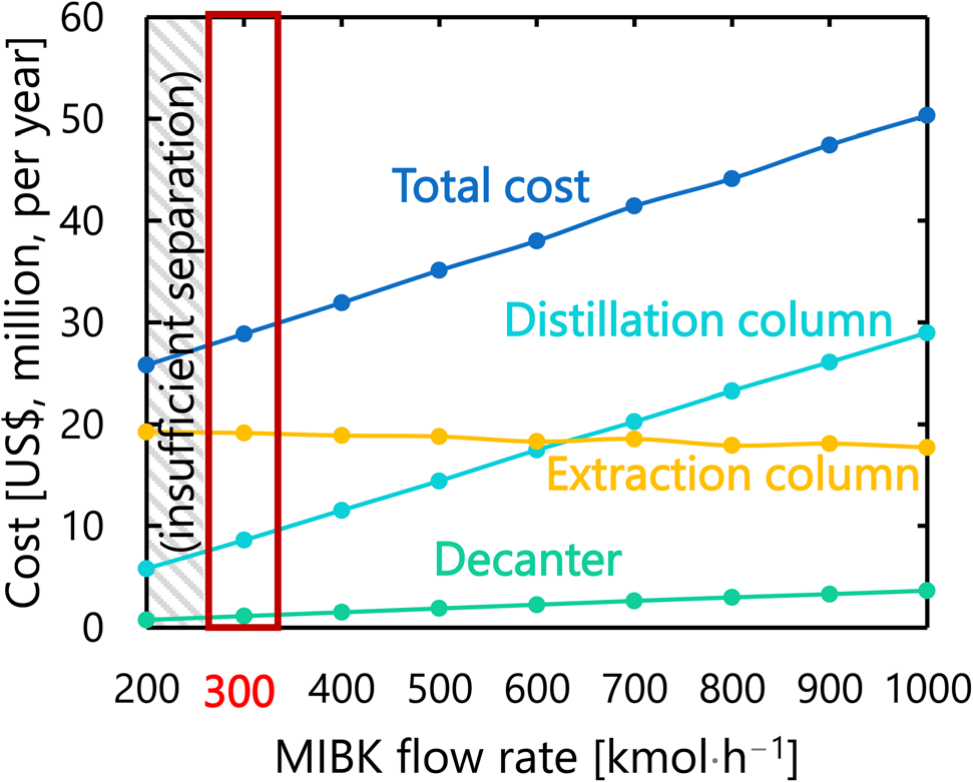
Dependence of the extraction column cost, decanter cost, distillation column cost, and total cost on the flow rate of MIBK entering the extraction column. MIBK is methyl isobutyl ketone.

For the glucose separation process excluding the flash distiller (*i.e.*, the extraction column, decanter, and distillation column), Fig. 12 presents the dependence of the extraction column cost, decanter cost, distillation column cost, and total cost on the flow rate of MIBK entering the extraction column. It was observed that, as the flow rate of MIBK flowing into the extraction column increases, the extraction column cost decreases while the decanter cost and distillation column cost increase. As a result, the optimal flow rate of MIBK entering the extraction column was found to be 300 kmol L^−1^, to minimize the total cost. The optimized design of the glucose separation process excluding the flash distiller, namely the extraction column, decanter, and distillation column, is summarized in Tables 8–10, respectively.

**Table:** Summary of the optimal design of the extraction column in the glucose separation process

Number of trays	10
Column height	7.00 m
Column diameter	2.40 m
Column pressure	3.5 bar

**Table:** Summary of the optimal design of the decanter in the glucose separation process

Decanter pressure	1.0 bar
Decanter temperature	25 °C
Decanter height length	4.65 m
Decanter diameter	1.16 m

**Table:** Summary of the optimal design of the distillation column in the glucose separation process

Column pressure	1.0 bar
Number of trays	5
Number of the feeding tray	2
Reflux ratio	0.50
Column height	8.20 m
Column diameter	3.16 m
Condenser temperature	94.7 °C
Removed heat	18.5 GJ h^−1^
Reboiler temperature	320.1 °C
Supplied heat	24.9 GJ h^−1^

### 3.3. Hydrogen production process

**Fig. 13:**
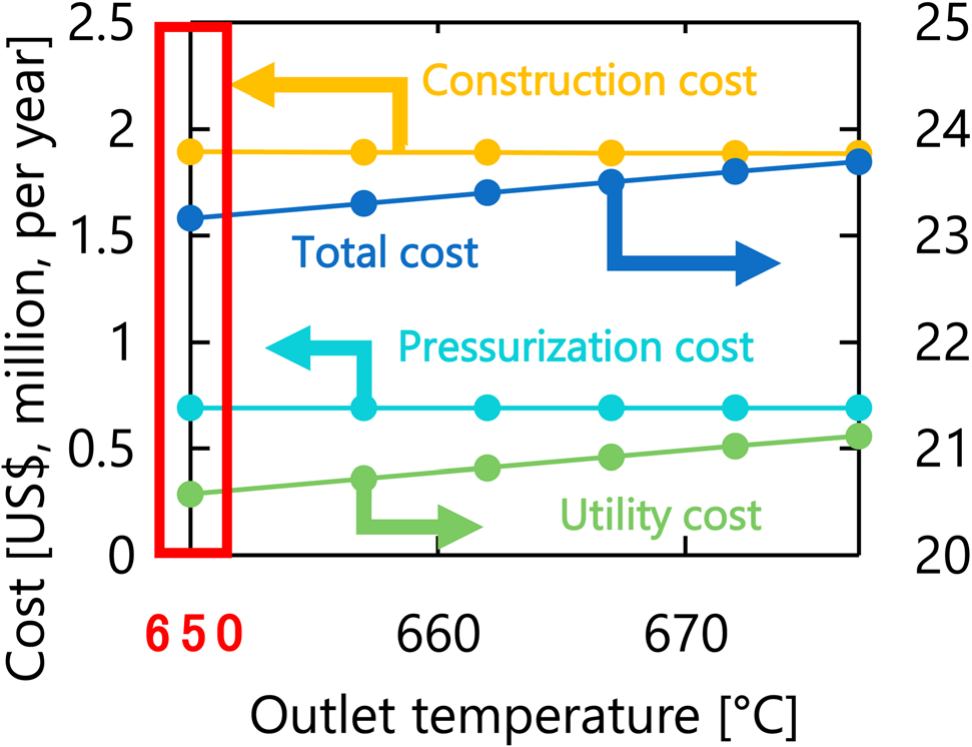
Dependence of the construction cost, utility cost, pressurization cost, and total cost on the outlet temperature of the hydrogen production reactor, with a fixed pressure of 26 MPa.

**Fig. 14:**
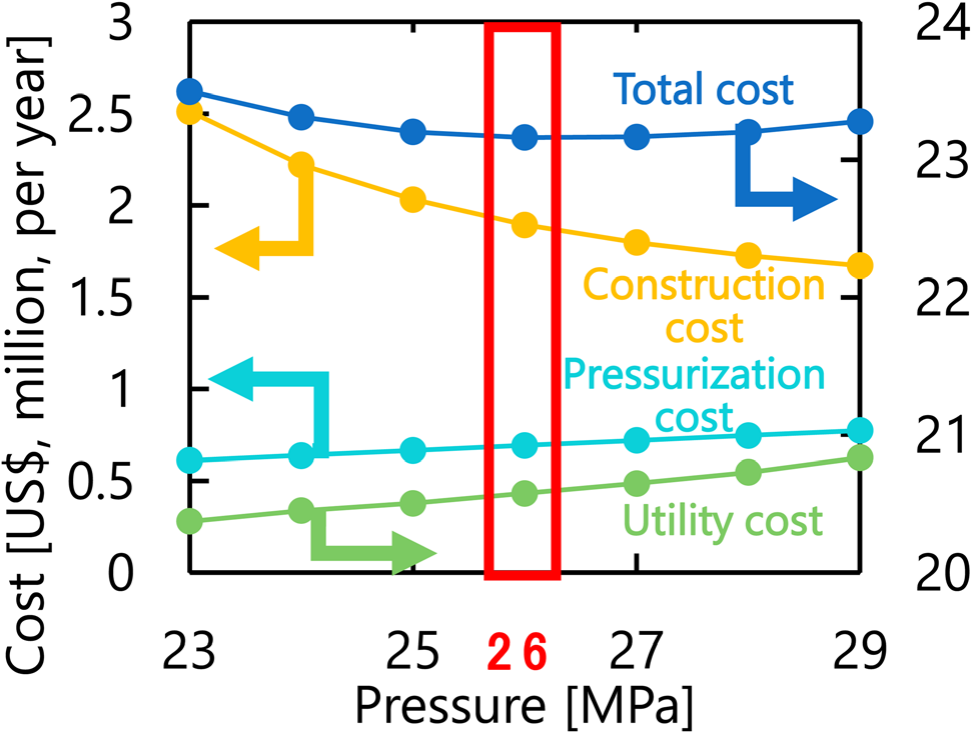
Dependence of the construction cost, utility cost, pressurization cost, and total cost on the pressure of the hydrogen production reactor, with a fixed outlet temperature of 650 °C.

**Fig. 15:**
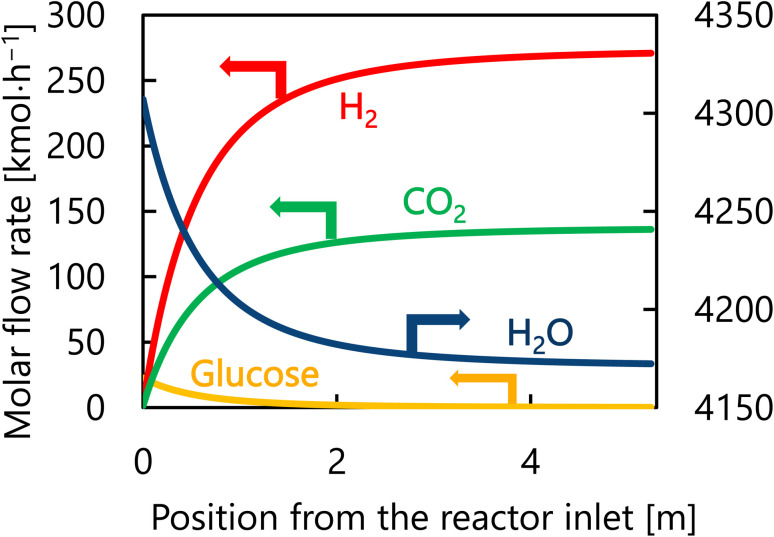
Spatial profile of the molar flow rate of each component of each component in the hydrogen production reactor for the optimized condition.

Let us move on to the calculation results for the hydrogen production process. Fig. 13 presents the dependence of the construction cost, utility cost, pressurization cost, and total cost on the outlet temperature of the hydrogen production reactor, with a fixed pressure of 26 MPa. As the outlet temperature increases, the construction cost becomes smaller because the required reactor volume is smaller due to the higher reaction rate instead of the larger utility cost. As a result, the optimum outlet temperature of the hydrogen production reactor was found to be 650 °C. Fig. 14 presents the dependence of the construction cost, utility cost, pressurization cost, and total cost on the pressure of the hydrogen production reactor, with a fixed outlet temperature of 650 °C. As the pressure in the reactor increases, the pressurization cost and utility cost increase, and the construction cost decreases. The optimal reactor pressure was consequently observed to be 26 MPa. The optimized design of the hydrogen production process is summarized in Table 11. As a reference, the spatial profile of the molar flow rate of each component in the hydrogen production reactor for the optimized condition is presented in Fig. 15.

**Table:** Summary of the optimal design of the hydrogen production reactor. PFR stands for plug flow reactor

Reactor type	PFR
Reactor length	5.32 m
Reactor diameter	1.77 m
Glucose conversion	0.99
Reactor pressure	26 MPa
Inlet line velocity	0.21 m s^−1^
Inlet temperature	667 °C
Outlet temperature	650 °C

### 3.4. Hydrogen separation process

**Fig. 16:**
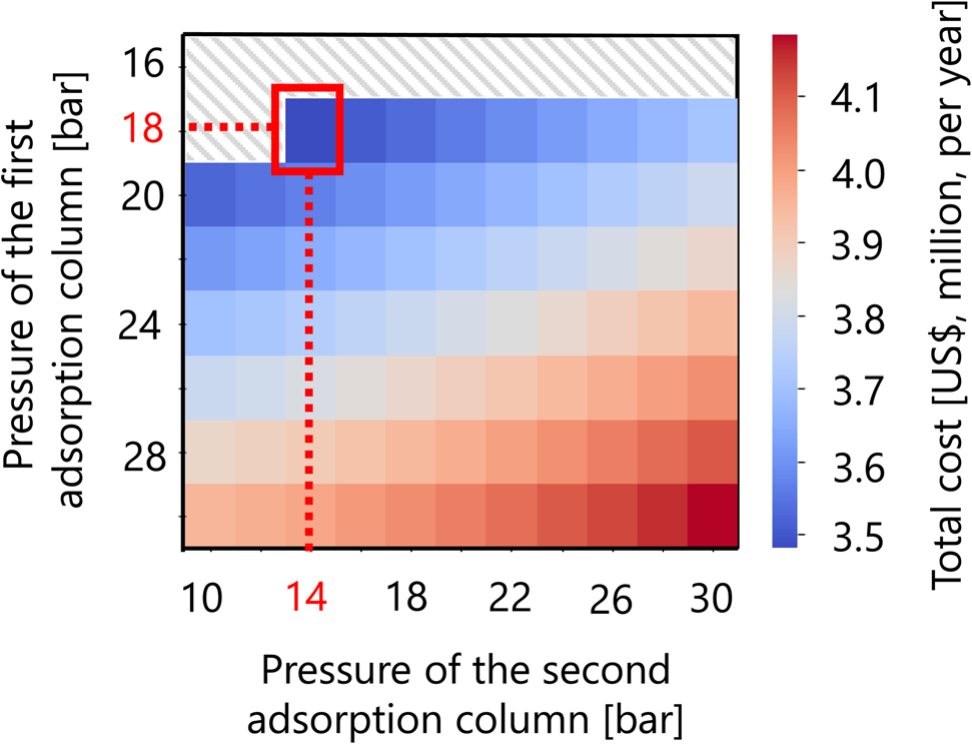
Dependence of the total cost on the pressures of the first and second adsorption columns of the PSA process. The total cost refers to the sum of hydrogen loss cost, construction cost, and adsorbent cost.

For the hydrogen separation process, firstly, the optimized design of the gas–liquid separator is summarized in Table 12. Secondly, Fig. 16 presents the calculation result of the dependence of the total cost on the pressures of the first and second adsorption columns of the PSA process. The total cost in this part refers to the sum of hydrogen loss cost, construction cost, and adsorbent cost, as described in Section 2.5. It was observed that the smaller the PSA adsorption column pressure, the more the adsorbent cost increases, while the hydrogen loss cost and construction cost decrease. As a result, the optimal pressures of the first and second PSA adsorption columns were found to be 18 and 14 bar, respectively. The cost breakdown for the first PSA column was that the hydrogen loss cost, construction cost, and adsorbent cost were 98.1%, 1.7%, and 0.58%, respectively ($1.87M per year in total). For the second PSA column, they were 98.4%, 1.3%, and 0.20%, respectively ($1.61M per year in total). Thus, it was observed that the hydrogen loss cost is dominant in the total cost for both columns. The optimized design of the PSA columns in the hydrogen separation process is summarized in Table 13.

**Table:** Summary of the optimal design of the gas–liquid separator in the hydrogen separation process

Separator pressure	30.0 bar
Separator temperature	25 °C
Column height	1.0 m
Column diameter	0.5 m

**Table:** Summary of the optimal design of the PSA columns in the hydrogen separation process. PSA is pressure swing adsorption

	1st PSA column	2nd PSA column
Column diameter	0.90 m	0.92 m
Column height	3.57 m	2.49 m
Number of columns	2	2
Adsorption column pressure	18 bar	14 bar
Desorption column pressure	0.2 bar	0.2 bar
Operation temperature	25 °C	25 °C
Superficial velocity	0.18 m s^−1^	0.18 m s^−1^
Switchover time	300 s	300 s

### 3.5. Levulinic acid separation process

As described in Section 2.6, the levulinic acid separation process is consist of three consecutive units: a flash distiller, decanter, and distillation column. The design of these separation units optimized based on the total cost for the target hydrogen production amount is summarized in Tables 14–16, respectively.

**Table:** Summary of the optimal design of the flash distiller in the levulinic acid separation process

Distiller pressure	0.5 bar
Distiller temperature	500 °C
Column height	0.48 m
Column diameter	0.24 m

**Table:** Summary of the optimal design of the decanter in the levulinic acid separation process

Decanter pressure	1.0 bar
Decanter temperature	100 °C
Decanter length	0.94 m
Decanter diameter	0.23 m

**Table:** Summary of the optimal design of the distillation column in the levulinic acid separation process

Column pressure	1.0 bar
Number of trays	5
Number of the feeding tray	3
Reflux ratio	0.30
Column height	8.20 m
Column diameter	0.14 m
Condenser temperature	81.8 °C
Removed heat	0.1 GJ h^−1^
Reboiler temperature	256 °C
Supplied heat	0.1 GJ h^−1^

### 3.6. Entire process flow and heat integration

**Fig. 17:**
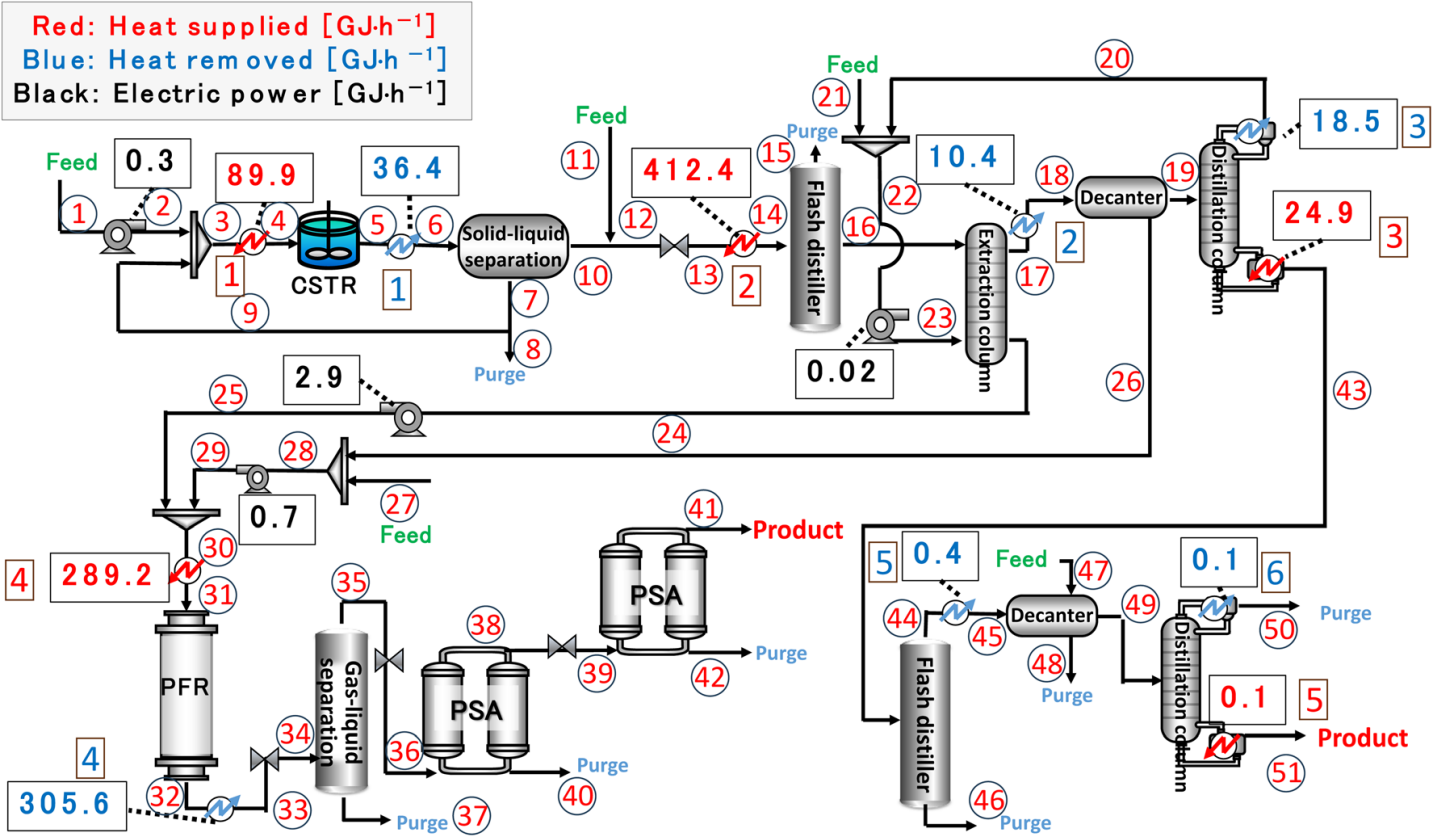
Process flow diagram of the resulting entire process with heat balance. The numbers in circles and squares labelled at positions denote the position numbers used in Tables 17–19, respectively. CSTR is continuous stirred-tank reactor, PFR is plug flow reactor, and PSA is pressure swing adsorption.

**Fig. 18:**
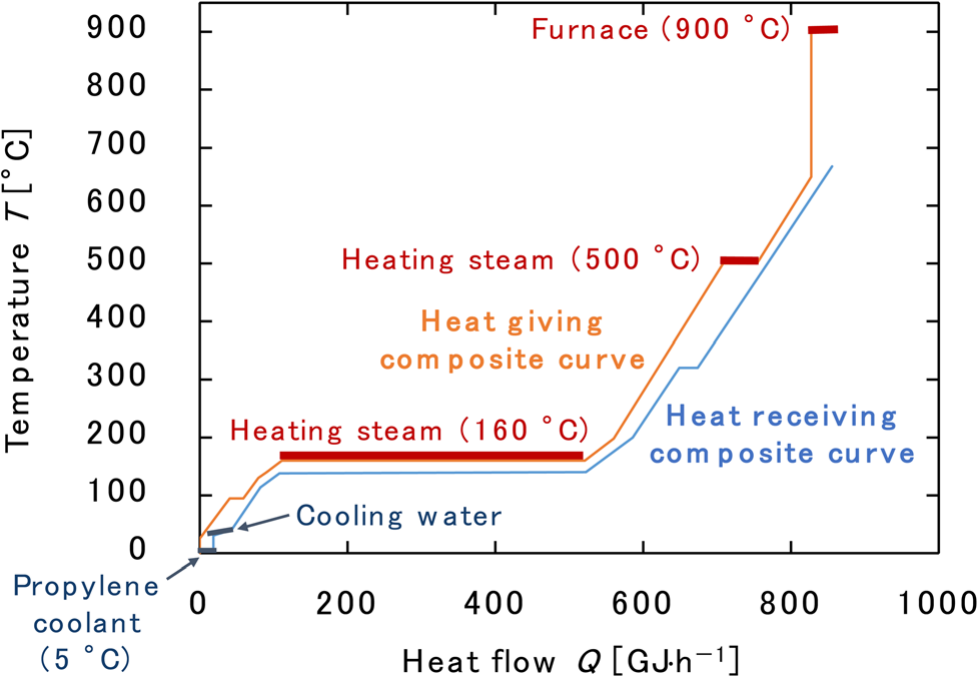
Composite curves for the entire process. The minimum approach temperature difference was set to 10 °C.

Finally, the process flow diagram of the resulting entire process with heat balance is presented in Fig. 17. The mass balance sheet of the process is presented as Table 17. For heat exchange, the temperature change of the heat giving fluid and the heat given are shown in Table 18, and the temperature change of the heat receiving fluid and the heat received are shown in Table 19. The composite curves,^9,56,57^ also known as temperature–enthalpy (*T*–*Q*) diagram, for the entire process are drawn in Fig. 18. The minimum approach temperature difference was set to 10 °C. As a result of heat integration based on Fig. 18, the utility cost was reduced from $68.9M per year to $33.2M per year.

Mass balance sheet of the resulting entire process. The numbers labelled in the first line of the table denote the position numbers in circles in Fig. 17. The temperature and pressure at each position are accompanied. HMF is hydroxymethylfurfural and MIBK is methyl isobutyl ketone

**Table:** 

[kmol h^−1^]	1	2	3	4	5	6	7	8	9	10
Cellulose	33.0	33.0	66.1	66.1	34.9	34.9	34.9	1.7	33.1	0.0
Glucose	0.0	0.0	0.2	0.2	23.0	23.0	0.3	0.01	0.2	22.8
HMF	0.0	0.0	0.021	0.02	1.9	1.9	0.02	0.001	0.02	1.9
Levulinic acid	0.0	0.0	0.03	0.03	3.1	3.1	0.03	0.001	0.03	3.1
Formic acid	0.0	0.0	0.03	0.03	3.1	3.1	0.03	0.001	0.03	3.1
Humin	0.0	0.0	67.1	67.1	70.6	70.6	70.6	3.5	67.1	0.0
H_2_O	0.0	0.0	13880.4	13880.4	13857.9	13857.9	153.1	7.7	145.5	13704.8
H_2_SO_4_	75.4	75.4	76.2	76.2	76.2	76.2	0.8	0.04	0.8	75.3
Na_2_SO_4_	0.0	0.0	0.0	0.0	0.0	0.0	0.0	0.0	0.0	0.0
NaOH	0.0	0.0	0.0	0.0	0.0	0.0	0.0	0.0	0.0	0.0
MIBK	0.0	0.0	0.0	0.0	0.0	0.0	0.0	0.0	0.0	0.0
H_2_	0.0	0.0	0.0	0.0	0.0	0.0	0.0	0.0	0.0	0.0
CO_2_	0.0	0.0	0.0	0.0	0.0	0.0	0.0	0.0	0.0	0.0
Flow rate [kg h^−1^]	12745.8	12745.8	280382.3	280382.3	280391.1	280391.1	21275.8	1050.9	20198.4	259131.0
Temperature [°C]	25.0	25.2	31.5	200.0	198.0	130.0	130.0	130.0	130.0	130.0
Pressure [bar]	1.0	16.0	16.0	16.0	16.0	16.0	16.0	16.0	16.0	16.0

**Table:** 

[kmol h^−1^]	11	12	13	14	15	16	17	18	19	20
Cellulose	0.0	0.0	0.0	0.0	0.0	0.0	0.0	0.0	0.0	0.0
Glucose	0.0	22.8	22.8	22.8	0.0	22.8	0.9	0.9	0.1	0.0
HMF	0.0	1.9	1.9	1.9	0.0	1.9	1.9	1.9	1.9	0.0
Levulinic acid	0.0	3.1	3.1	3.1	0.6	2.4	2.3	2.3	2.2	0.0
Formic acid	0.0	3.1	3.1	3.1	1.6	1.5	0.1	0.1	0.0	0.0
Humin	0.0	0.0	0.0	0.0	0.0	0.0	0.0	0.0	0.0	36.1
H_2_O	0.0	13855.5	13855.5	13855.5	10500.5	3355.0	125.4	125.4	36.1	285.4
H_2_SO_4_	0.0	0.0	0.0	0.0	0.0	0.0	0.0	0.0	0.0	0.0
Na_2_SO_4_	0.0	75.3	75.3	75.3	0.0	75.3	14.6	14.6	11.9	0.0
NaOH	150.6	0.0	0.0	0.0	0.0	0.0	0.0	0.0	0.0	0.0
MIBK	0.0	0.0	0.0	0.0	0.0	0.0	287.0	287.0	286.4	285.4
H_2_	0.0	0.0	0.0	0.0	0.0	0.0	0.0	0.0	0.0	0.0
CO_2_	0.0	0.0	0.0	0.0	0.0	0.0	0.0	0.0	0.0	0.0
Flow rate [kg h^−1^]	2564.9	264410.7	264410.7	264410.7	189312.8	75086.3	33606.3	33606.3	31417.7	40233.2
Temperature [°C]	25.0	137.8	137.8	140.0	140.0	140.0	139.8	25.0	25.0	94.7
Pressure [bar]	16.0	16.0	3.5	3.5	3.5	3.5	3.5	1.0	1.0	1.0

**Table:** 

[kmol h^−1^]	21	22	23	24	25	26	27	28	29	30
Cellulose	0.0	0.0	0.0	0.0	0.0	0.0	0.0	0	0	0.0
Glucose	0.0	0.0	0.0	21.9	21.9	0.8	0.0	0.8	0.8	22.7
HMF	0.0	0.0	0.0	0.0	0.0	0.0	0.0	0.03	0.03	0.0
Levulinic acid	0.0	0.0	0.0	0.1	0.1	0.1	0.0	0.09	0.09	0.2
Formic acid	0.0	0.0	0.0	1.5	1.5	0.0	0.0	0.04	0.04	1.5
Humin	0.0	0.0	0.0	0.0	0.0	0.0	0.0	0	0	0.0
H_2_O	0.0	36.1	36.1	3265.6	3265.6	89.4	952.0	1041.4	1041.4	4307.0
H_2_SO_4_	0.0	0.0	0.0	0.0	0.0	0.0	0.0	0	0	0.0
Na_2_SO_4_	0.0	0.0	0.0	60.8	60.8	2.7	0.0	2.7	2.7	63.5
NaOH	0.0	0.0	0.0	0.0	0.0	0.0	0.0	0	0	0.0
MIRK	14.6	299.9	299.9	13.0	13.0	0.5	0.0	0.5	0.5	13.6
H_2_	0.0	0.0	0.0	0.0	0.0	0.0	0.0	0	0	0.0
CO_2_	0.0	0.0	0.0	0.0	0.0	0.0	0.0	0	0	0.0
Flow rate [kg h^−1^]	1462.3	30690.6	30690.6	72193.0	72193.0	2177.6	17150.5	19328.2	19328.2	91530.5
Temperature [°C]	25.0	91.6	91.9	125.6	134.9	25.0	25.0	25.0	33.6	113.6
Pressure [bar]	1.0	1.0	3.5	3.5	260.0	1.0	1.0	1.0	260.0	260.0

**Table:** 

[kmol h^−1^]	31	32	33	34	35	36	37	38	39	40
Cellulose	0.0	0.0	0.0	0.0	0.0	0.0	0.0	0.0	0.0	0.0
Glucose	22.7	0.2	0.2	0.2	0.0	0.0	0.2	0.0	0.0	0.0
HMF	0.0	0.0	0.0	0.0	0.0	0.0	0.0	0.0	0.0	0.0
Levulinic acid	0.2	0.2	0.2	0.2	0.0	0.0	0.2	0.0	0.0	0.0
Formic acid	1.5	0.0	0.0	0.0	0.0	0.0	0.0	0.0	0.0	0.0
Humin	0.0	0.0	0.0	0.0	0.0	0.0	0.0	0.0	0.0	0.0
H_2_O	4307.0	4172.3	4172.3	4172.3	0.3	0.3	4172.0	0.3	0.3	0.0
H_2_SO_4_	0.0	0.0	0.0	0.0	0.0	0.0	0.0	0.0	0.0	0.0
Na_2_SO_4_	63.5	63.5	63.5	63.5	0.0	0.0	63.5	0.0	0.0	0.0
NaOH	0.0	0.0	0.0	0.0	0.0	0.0	0.0	0.0	0.0	0.0
MIRK	13.6	13.6	13.6	13.6	0.0	0.0	13.5	0.0	0.0	0.0
H_2_	0.0	270.9	270.9	270.9	253.5	253.5	17.4	240.2	240.2	13.3
CO_2_	0.0	136.2	136.2	136.2	46.3	46.3	89.9	1.0	1.0	45.3
Flow rate [kg h^−1^]	91530.5	91521.5	91521.5	91521.5	2558.1	2558.1	88957.4	537.6	537.6	2020.5
Temperature [°C]	667.0	650.0	25.0	25.0	25.0	25.0	25.0	25.0	25.0	25.0
Pressure [bar]	260.0	260.0	260.0	30.0	30.0	18.0	30.0	18.0	14.0	0.2

**Table:** 

[kmol h^−1^]	41	42	43	44	45	46	47	48	49	50	51
Cellulose	0.0	0.0	0.0	0.0	0.0	0.0	0.0	0.0	0.0	0.0	0.0
Glucose	0.0	0.0	0.1	0.1	0.1	0.0	0.0	0.1	0.0	0.0	0.0
HMF	0.0	0.0	1.9	1.9	0.0	1.9	0.0	0.0	0.0	0.0	0.0
Levulinic acid	0.0	0.0	2.2	2.2	2.2	0.1	0.0	0.2	1.9	0.0	1.9
Formic acid	0.0	0.0	0.0	0.0	0.0	0.0	0.0	0.0	0.0	0.0	0.0
Humin	0.0	0.0	0.0	0.0	0.0	0.0	0.0	0.0	0.0	0.0	0.0
H_2_O	0.3	0.0	0.0	0.0	0.0	0.0	4.0	3.6	0.4	0.4	0.0
H_2_SO_4_	0.0	0.0	0.0	0.0	0.0	0.0	0.0	0.0	0.0	0.0	0.0
Na_2_SO_4_	0.0	0.0	11.9	0.0	0.0	11.9	0.0	0.0	0.0	0.0	0.0
NaOH	0.0	0.0	0.0	0.0	0.0	0.0	0.0	0.0	0.0	0.0	0.0
MIRK	0.0	0.0	1.0	0.6	0.6	0.5	0.0	0.0	0.6	0.6	0.0
H_2_	228.7	11.5	0.0	0.0	0.0	0.0	0.0	0.0	0.0	0.0	0.0
CO_2_	0.0	0.9	0.0	0.0	0.0	0.0	0.0	0.0	0.0	0.0	0.0
Flow rate [kg h^−1^]	471.3	62.8	2180.3	328.1	328.1	1871.8	74.1	102.9	289.7	69.1	220.6
Temperature [°C]	25.0	25.0	320.1	500.0	100.0	500.0	25.0	100.0	100.0	81.8	250.6
Pressure [bar]	14.0	0.2	1.0	0.5	1.0	0.5	1.0	1.0	1.0	1.0	1.0

**Table:** Temperature change of the heat giving fluid and the heat given for heat exchange. The number labelled for each heat giving fluid denotes each position number in a square labelled in Fig. 17

Heat giving fluid	*T*_in_ [°C]		*T*_out_ [°C]	Heat flow [GJ h^−1^]
# 1	198.0	→	130.0	36.4
# 2	139.8	→	25.0	10.4
# 3	94.7	→	94.7	18.5
# 4	650.0	→	25.0	305.6
# 5	500.0	→	100.0	0.4
# 6	81.8	→	81.8	0.1
Steam	160	→	160	410.1
Steam	500	→	500	23.0
Furnace	900	→	900	28.3

**Table:** Temperature change of the heat receiving fluid and the heat received for heat exchange. The number labelled for each heat receiving fluid denotes each position number in a square labelled in Fig. 17

Heat receiving fluid	*T*_in_ [°C]		*T*_out_ [°C]	Heat flow [GJ h^−1^]
# 1	31.5	→	200.0	89.9
# 2	137.8	→	140	412.4
# 3	320.6	→	320.6	24.9
# 4	113.6	→	667.0	289.2
# 5	250.6	→	250.6	0.1
Propylene coolant	5.0	→	5.0	18.5
Cooling water	30	→	40	20.0

### 3.7. Economic evaluation

Table 20 presents the breakdown of annual expenditures and annual revenues for the process. The plant depreciation period was set at 3 years only for the reactor in the glucose production process, considering the influence of corrosion, and 7 years for the other reactors. Eventually, the annual profit was −$26.9M per year (−$0.015 per mol-H_2_), which is negative. This might be regarded as the price we human have to pay for renewable hydrogen production from biomass at the present stage. Let us discuss the main causes of the deficit. The most significant issue in this process is that dilute cellulose solution is reacted under sulfuric acid conditions in the glucose production process. We simply adopted the experimental condition in ref. 26 in our present study. Namely, the cellulose solution concentration was 4 wt% and the sulfuric acid concentration was 0.3 mol L^−1^, which required a large amount of water and sulfuric acid. This necessitated a neutralizer cost for neutralizing the sulfuric acid and a utility cost for removing a large amount of water in the glucose separation process. Also, the more water that flows in after flash distillation in the glucose separation process, the higher the cost for newly required extractant. As a result, the neutralizer cost to neutralize sulfuric acid was $14.4M per year, the utility cost to remove water was $23.6M per year, and the extractant cost to extract glucose was $18.9M per year. These costs dominated as large as 72% of the total expenditures. Therefore, to improve the economic performance of the process, it is necessary to consider the reaction of cellulose solution at a higher concentration to reduce the burden of glucose separation. In addition, the hydrogen exhausted from the bottom of the second PSA column was of low purity, about 95 vol%, and could not be sold as a product. One way to make effective use of this hydrogen exhaust is to use another PSA to increase the purity of hydrogen for commercialization, or to reduce the cost of fuel hexane by feeding the hydrogen into a heating furnace. It should be noted that the basic prices of chemicals often significantly fluctuate due to global circumstances, and therefore our cost calculation may carry a potential margin of error of up to 50%. In addition, it should be noted that the separation cost is included in the price of cellulose, as mentioned in Section 2.7, which contributes to the deficit. If the raw materials containing lignin and hemicellulose, for instance, could be properly processed, the economics could be improved from lower raw material prices and the sale of byproducts.

**Table:** Breakdown of the annual expenditures and annual revenues for the process. MIBK is methyl isobutyl ketone

Expenditure		[$ per year]
Raw material cost	MIBK (extractant)	18.9M
	NaOH (neutralizer)	14.4M
	Cellulose	4.2M
	Sulfuric acid	2.9M
	Water	0.5M
	Activated carbon	0.008M
Construction cost	Reactors	2.6M
	Separators	0.7M
	Pressure transformers	0.4M
	Heat exchangers	0.3M
	Furnaces	0.2M
Utility cost	Fuel hexane	2.2M
	Electric power	0.8M
	Steam	29.3M
	Cooling water	0.08M
	Propylene coolant	0.7M
Labor cost		0.6M
Total		78.8M
Revenue		[$ per year]
	Hydrogen	34.0M
	Levulinic acid	17.9M
Total		51.9M

### 3.8. Comparison with other hydrogen production schemes

Table 21 presents a comparison of the so-called green, blue, gray, and yellow hydrogen in terms of energy, CO_2_ emissions, and economic cost required for the hydrogen production of 41 million Nm^3^ per year (*i.e.*, 228.7 kmol h^−1^). Green hydrogen is hydrogen produced using renewable energy, and is classified as water electrolysis using electricity generated by wind power^58^ and biomass-derived hydrogen production.^59^ Blue hydrogen is classified as hydrogen produced by steam reforming of methane, a production method in which the emitted CO_2_ is recovered.^60^ Gray hydrogen is classified by steam reforming of methane, where hydrogen is produced but no CO_2_ is recovered.^60^ Yellow hydrogen is classified as electrolysis of water using electricity from the power grid.^60^ The details of the calculation methods of the evaluation parameters for each type of hydrogen are presented in the ESI.† Note that for the category of electrolysis of water by wind power, only the electrolysis process was considered for simplicity.

Comparison of the green, blue, gray, and yellow hydrogen in terms of energy, CO_2_ emissions, and economic cost required for the hydrogen production of 41 million Nm^3^ per year (228.7 kmol h^−1^)

**Table:** 

	Green hydrogen				Blue hydrogen
	Decomposition of cellulose (this study)	Electrolysis of water by wind power (electrolysis only, experimental)	Electrolysis of water by wind power (electrolysis only, theoretical)	Electrolysis of water by wind power (reaction enthalpy)	Steam reforming of methane (w/CO_2_ capture)
Energy [GJ h^−1^]	32.1	439.1	441.2	54.89	87.5
Energy [MJ mol^−1^-H_2_]	0.140	1.92	1.93	0.240	0.383
CO_2_ emission [kmol h^−1^]	228.9	6.097	6.126	3.811	10.3
CO_2_ emission [mol-CO_2_/mol-H_2_]	1.00	0.0267	0.0268	0.0167	0.0450
Cost [US$ per year]	78.8M	25.1M	46.7M	17.7M	7.37M
Cost [US$/mol-H_2_]	0.043	0.014	0.026	0.0097	0.0040

**Table:** 

	Gray hydrogen		Yellow hydrogen		
	Steam reforming of methane (w/o CO_2_ capture)	Steam reforming of methane (reaction enthalpy)	Electrolysis of water by electric power system (electrolysis only, experimental)	Electrolysis of water by electric power system (electrolysis only, theoretical)	Electrolysis of water by electric power system (reaction enthalpy)
Energy [GJ h^−1^]	72.3	18.3	209.1	210.1	54.89
Energy [MJ mol^−1^-H_2_]	0.316	0.0801	0.914	0.919	0.240
CO_2_ emission [kmol h^−1^]	94.2	67.6	215	216	195
CO_2_ emission [mol-CO_2_/mol-H_2_]	0.412	0.296	0.940	0.944	0.853
Cost [US$ per year]	5.09M	8.852M	25.1M	46.7M	17.7M
Cost [US$/mol-H_2_]	0.0028	0.0048	0.014	0.026	0.0097

For the energy required for hydrogen production, overall, our hydrogen production process from cellulose with supercritical water gasification requires less energy than other hydrogen production. This result can be attributed to the originally high enthalpy of cellulose and to the efficient heat exchange and effective use of heat in the process, as observed in Section 3.6. In fact, the energy required is 0.0731 times less than that of electrolysis of water by wind power (electrolysis process only), which is also green hydrogen, and 0.37 times less than that of steam reforming of methane (with CO_2_ capture), which is blue hydrogen. It is also 0.44 times less than that of steam reforming of methane (without CO_2_ capture), which is gray hydrogen, and 0.15 times less than that of the water electrolysis by the electric power system, which is yellow hydrogen. Thus, hydrogen production by our process, which is green hydrogen, is more energy-efficient than other hydrogen production methods.

For the amount of CO_2_ emission, we quantified the total CO_2_ emissions by combining both direct and indirect emissions, as outlined in Section S2 of the ESI.† For the nominal CO_2_ emission amount accompanied with hydrogen production, our process requires a larger amount of CO_2_ emissions than other hydrogen production. This is because, due to the stoichiometry of the reaction, our process produces more CO_2_ for every 1 mol of H_2_. For example, steam reforming of methane produces 0.25 mol of CO_2_ per mol of H_2_, while our process produces 0.5 mol of CO_2_ per mol of H_2_. Compared to electrolysis of water by wind power (green hydrogen), the required CO_2_ emissions are 37.5 times larger and compared to steam reforming of methane (blue), 22.2 times larger. Even compared to steam reforming of methane (gray), it counts 2.43 times larger and compared to electrolysis of water by the power system (yellow), 1.06 times larger. Nevertheless, since hydrogen from our process is derived from biomass, CO_2_ emissions may be considered zero because CO_2_ is absorbed through photosynthesis during the biomass growth process.

For the economic cost required for hydrogen production, it can be seen that our process is more costly than other hydrogen production. This result is mainly due to the neutralizer cost and the extractant cost, since the energy required is less compared to other hydrogen production methods as observed above, although the economic evaluation in Section 3.7 shows that the utility cost, neutralizer cost, and extractant cost account for most of the cost. The reason for the high neutralizer and extractant costs is that the glucose production process involves the reaction of dilute cellulose solution under sulfuric acid conditions, as discussed in Section 3.7. Since a large amount of water and sulfuric acid are required, the neutralizer cost for neutralization is considered to be high, and the extractant cost for glucose separation and extraction is also considered to be high. Compared to the electrolysis of water by wind power (green), the economic cost required is 3.12 times higher and compared to steam reforming of methane (blue), 10.7 times higher. Compared to steam reforming of methane (gray), it costs 15.4 times higher and compared to the electrolysis of water by the electric power system (yellow), 3.12 times higher. Note that the cost for the electrolysis of water by wind power and for the electrolysis of water by the electric power system appear to be equal to each other because we accounted for only the cost from the electrolysis process but not the wind turbine and so forth for the electrolysis of water by the electric power system. Overall, for the conditions we assumed in our calculations, our process economically costs higher than the other hydrogen production methods. Nevertheless, our cost calculation carried out for the other methods are highly simplified and thus may not be a very fair comparison. Furthermore, the huge discrepancy between the triumph in the energy cost and the defeat in the economic cost implies a large room for the improvement of process details and economics of the biomass-based green hydrogen production.

## 4. Conclusions

In this study, a chemical process was designed to produce glucose as an intermediate product from cellulose as a raw material, and to produce 41 million Nm^3^ of hydrogen as the main product at 99.99 vol% purity per year and 1800 tons of levulinic acid as a co-product at 99 wt% purity per year using supercritical water gasification technology. In this design, a continuous tank reactor was employed because the reaction in the glucose production process involves solids, and using a tube-type reactor may clog the reactor with solids. In the glucose separation process, glucose and levulinic acid, which cannot be separated by boiling point difference, were separated by using an extraction column. In the hydrogen separation process, the hydrogen purity, which could not be sufficiently increased with a single PSA process, was increased to the target value by employing two sets of PSA columns. The overall utility cost was significantly reduced through heat integration. Our economic evaluation for this process concluded that the annual profit would be −$26.9M per year (−$0.015/mol-H_2_), which is negative, as a price to be paid by the human for the renewable hydrogen production from biomass. By simply adopting the experimental condition in ref. 26, our chemical process contains a large amount of water and sulfuric acid, which requires an enormous cost for the neutralizer, drying utility, and extractant. To improve the economic performance of the process, it is necessary to consider the reaction of cellulose solution at a higher concentration to reduce the burden of glucose separation. In addition, the effective use of the wasted hydrogen with a purity of about 95 vol% from the second PSA column may also improve the process economics. As we compared the energy costs and economics of our process with other various representative hydrogen production schemes, the energy required for hydrogen production was found to be significantly smaller in our process, but the economic cost was considerably higher. This contradicting situation suggests a significant opportunity for enhancing the process details and economics of biomass-based green hydrogen production.

## Conflicts of interest

The authors declare that they have no known competing financial interests or personal relationships that could have appeared to influence the work reported in this paper.

## Supplementary Material

RA-013-D3RA05367A-s001
