# Supplementary material for: Proposal, design, and cost analysis of a hydrogen production process from cellulose via supercritical water gasification
Source: RSC Adv. 2023 Oct 16;13(43):30306–28. doi: 10.1039/d3ra05367a (PMC10577642; doi:10.1039/d3ra05367a)
Supplement: RA-013-D3RA05367A-s001 [file RA-013-D3RA05367A-s001.pdf]

Electronic Supplementary Material (ESI) for RSC Advances.
This journal is © The Royal Society of Chemistry 2023

Proposal, design, and cost analysis of a hydrogen production process from cellulose via supercritical water gasification

Supplementary Information

Taichi Masuda, Naoki Ikesaka, Yosuke Muranaka, and Katsuaki Tanabe*

Department of Chemical Engineering, Kyoto University, Nishikyo, Kyoto 615-8510, Japan

*e-mail: tanabe@cheme.kyoto-u.ac.jp

S1. Required Energy

S1-1. Decomposition of cellulose (This study)

Let us determine the energy required by our hydrogen production process. As observed in Section 3-6, the minimum heating value is 28.3 GJ/h. In addition to this, the electricity used in this process is 3.76 GJ/h. Therefore, the amount of energy required for our process can be calculated as 28.3 + 3.76 = 32.1 GJ/h.

S1-2. Electrolysis of water by wind power (Electrolysis only, experimental)

Let us consider the electrolysis of water when using an electrolysis tank to produce 42 m^3/h of hydrogen gas with 200 kW of electricity [S1], so that when 228.7 kmol/h of hydrogen is produced, the volume of hydrogen can be calculated as follows if hydrogen is an ideal gas.

(Volume of H_2 [m^3/h]) = (H_2 production [mol/h]) × (volume of ideal gas [m^3/mol])

= $228.7\times10^3 \times 22.4\times0.1^3$

= 5.12×10^3 m^3/h

Since it takes 200 kJ/s per 42 m^3/h of hydrogen gas in a commercial electrolysis tank [35], the power [GJ/h] required to produce 5.12×10^3 m^3/h can be calculated as follows.

(Electric power [GJ/h] required)

= (H_2 target production [m^3/h])/(H_2 production [m^3/h] for commercial equipment) × (energy [GJ/h] for commercial equipment)

= $5123/42 \times 200\times10^3 \times 3600\times10^{-9}$

= 87.8 GJ/h

Here, if the conversion efficiency η = 0.20 [S2] for 87.8 GJ/h of electricity,

(Required energy [GJ/h]) = (Required electrical energy [GJ/h])/η

= 87.8/0.20 = 439.1 GJ/h

S1-3. Electrolysis of water by wind power (Electrolysis only, theoretical)

From the energy required for the electrolysis of water, calculate the energetic energy in the electrolysis of water. From the following half-reaction equation [S3], assuming the number of electrons n = 2 mol/mol, the potential E = 2.0 V [S3], and the conversion efficiency η = 0.20 [S2],

$2OH^- \rightarrow 1/2O_2 + H_2O + 2e^-$
$2H^+ + 2e^- \rightarrow H_2$
$\Delta G = nFE/\eta = (2\times96454.56\times2.0)/0.20 = 1929091$ J/mol
Hydrogen production is 228.7 kmol/h, so the amount of energy required per 1 h is
(Energy required for electrolysis of water [GJ/h])
= $\Delta G \times$ (H_2 production [mol/h])
= $1929091 \times 228.7\times10^3 \times 10^{-9}$
= 441.2 GJ/h

S1-4. Electrolysis of water by wind power (Reaction enthalpy)

Calculating the energy required for the electrolysis of water from the enthalpy of water production, 240 kJ/mol [S4],
(Energy required for H_2 production [GJ/h])
= (enthalpy of production of H_2O [GJ/mol]) × (H_2 production [mol/h])
= $240\times10^{-6} \times 228.7\times10^3$
= 54.89 GJ/h

S1-5. Steam reforming of methane (w/ CO_2 capture)

Calculate the energy required in the process of steam reforming of methane with carbon dioxide capture. Since the energy required to produce 8.994 t/h of hydrogen is 433.72 MW [S5], we can calculate the energy required to produce 228.7 kmol/h of hydrogen.
(Energy required for H_2 production [GJ/h]) = (Energy required per 1 mol/h of hydrogen [GJ/mol]) × (Hydrogen production [mol/h])
= $(433.72\times3600\times10^{-3}) / (8.994/2\times907\times10^3) \times (228.7\times10^3)$
= 87.5 GJ/h

S1-6. Steam reforming of methane (w/o CO_2 capture)

Calculate the energy required for the methane steam reforming process without carbon dioxide capture. Since the energy required to produce 8.994 t/h of hydrogen is 394.77 MW [S5], we can calculate the energy required to produce 228.7 kmol/h of hydrogen.
(Energy required for H_2 production [GJ/h]) = (Energy required per 1 mol/h of hydrogen

[GJ/mol]) × (Hydrogen production [mol/h])
= $(394.77 \times 3600 \times 10^{-3}) / (8.994/2 \times 10^{6}) \times (228.7 \times 10^{3})$
= 72.3 GJ/h

S1-7. Steam reforming of methane (Reaction enthalpy)

The energy required for hydrogen production by steam reforming of methane is calculated in terms of reaction enthalpy. The reaction equation for hydrogen production by steam reforming of methane is shown below [S6].

$$CH_4 + H_2O \rightarrow CO + 3H_2 \qquad \Delta H^{\circ}_{298} = 206 \text{ kJ/mol} \qquad \text{(S1-7-1)}$$

$$CO + H_2O \rightarrow CO_2 + H_2 \qquad \Delta H^{\circ}_{298} = -41 \text{ kJ/mol} \qquad \text{(S1-7-2)}$$

In addition, the temperature-dependent molar heat capacity at constant pressure of each substance extracted from Aspen Plus was used. First, since the reforming reaction of Eq. S1-7-1 takes place at a temperature of 973 K, the heat of reaction for the reforming reaction at a temperature of 973 K is determined as follows:
(Heat of reaction of the reforming reaction at temperature 973 K [kJ/mol]) = (standard heat of reaction of the reforming reaction [kJ/mol]) + (enthalpy of the standard state reference product at 973 K [kJ/mol]) − (enthalpy of the standard state reference reactant at 973 K [kJ/mol])
= 206 + (59.6+140) − (36.4+24.9)
= 344.3 kJ/mol

First, since the shift reaction in Eq. S1-7-2 takes place at a temperature of 573 K, the heat of reaction for the shift reaction at a temperature of 573 K is determined as follows:
(Heat of reaction of the shift reaction at temperature 573 K [kJ/mol]) = (standard heat of reaction of the shift reaction [kJ/mol]) + (enthalpy of product at 573 K of standard state reference [kJ/mol]) − (enthalpy of reactant at 573 K of standard state reference [kJ/mol])
= −41 + (8.02+11.7) − (55.2+9.58)
= −86.06 kJ/mol

Hence, the energy required to produce 228.7 kmol/h of hydrogen by steam reforming of methane can be obtained as follows:
(Energy required for H_2 production [GJ/h]) = (Amount of reactants [mol/h]) × (Reaction

heat of reforming reaction [GJ/mol]) + (Amount of reactants [mol/h]) × (Reaction heat of shift reaction [GJ/mol])
$= (228.7\times10^{3})/4 \times 344.3\times10^{-6}) - (228.7\times10^{3})/4 \times 86.06\times10^{-6})$
= 14.8 GJ/h

Next, find the energy required by the furnace to heat CH_4 and H_2O to 973 K, respectively. The energy required to heat CH_4 and H_2O to 973 K is 36.4 kJ/mol and 24.9 kJ/mol per mol, respectively, and can be calculated as follows.
(Energy required in heating furnace [GJ/h]) = (Amount of CH_4 [mol/h]) × (Energy required for CH_4 per mol [GJ/mol]) + (Amount of H_2O [mol/h]) × (Energy required for CH_4 per mol [GJ/mol])
$= (228.7\times10^{3})/4 \times 36.4\times10^{-6} + (228.7\times10^{3})/4 \times 24.9\times10^{-6}$
= 2.081 + 1.424
= 3.51 GJ/h
Hence, the energy required for hydrogen production is 14.8 + 3.51 = 18.3 GJ/h.

S1-8. Electrolysis of water by electric power system (Electrolysis only, experimental)

From Section S1-2, the electricity required to produce 228.7 kmol/h of hydrogen by electrolysis of water using an electrolysis tank is 87.8 GJ/h. Here, if the conversion efficiency of the power system is η = 0.42 [S7], the following can be obtained:
(Required amount of energy [GJ/h]) = (Required amount of electrical energy [GJ/h])/η
= 87.8/0.42 = 209.1 GJ/h

S1-9. Electrolysis of water by electric power system (Electrolysis only, theoretical)

It can be obtained in the same way as in Section S1-3. From the following semi-reaction equation [S3], assuming that the number of electrons n = 2 mol/mol, the potential E = 2.0 V [S3], and the conversion efficiency of the power system η = 0.42 [S7],
$2OH^{-} \rightarrow 1/2O_2 + H_2O + 2e^{-}$
$2H^{+} + 2e^{-} \rightarrow H_2$
$\Delta G = nFE/\eta = (2\times96454.56\times2.0)/0.42 = 918614.86$ J/mol
Hydrogen production is 228.7 kmol/h, so the amount of energy required per 1 h is
(Energy required for electrolysis of water [GJ/h])

= ΔG × (H_2 production [mol/h])
= 918614.86 × 228.7×10^3 × 10^{-9})
= 210.1 GJ/h

S1-10. Electrolysis of water by electric power system (Reaction enthalpy)

Same as Section S1-4.

S2. CO_2 Emission

S2-1. Decomposition of cellulose (This study)

Calculate the carbon dioxide emissions from our hydrogen production process. The carbon dioxide emissions from our process consist of the amount of carbon dioxide purged in the final flow shown in Fig. 3-6-1 and Table 3-6-1, and the amount of carbon dioxide emitted when the fuel, hexane, is burned in the furnace. The carbon dioxide purged is the sum of the carbon dioxide emitted in flow numbers 37, 40, and 42 in the final flow diagram in Fig. 3-6-1, which is 89.9 + 45.3 + 0.9 = 136.1 kmol/h. The energy required by the heating furnace is 28.3 GJ/h. Since the energy required to burn 1 kmol/h of hexane is 2031949 kJ/h based on Aspen HYSYS simulation results, the amount of hexane required is:
(Amount of hexane needed for the furnace [kmol/h]) = (Energy needed in the furnace [GJ/h]) / (Heat of combustion of hexane [GJ/kmol])
= 28.3 / (2031949×10^{-6})
= 13.9 kmol/h
Hence, since the molar ratio of hexane to carbon dioxide is six from the following reaction equation,
$2C_6H_{14} + 19O_2 \rightarrow 12CO_2 + 14H_2O$,
the amount of carbon dioxide emitted can be calculated as follows.
(Amount of carbon dioxide emitted from the furnace [kmol/h]) = (Amount of hexane burned [kmol/h]) × (Molar ratio of hexane to carbon dioxide)
= 13.93 × 6
= 83.6 kmol/h
Since 0.855 pound (lb) of carbon dioxide is emitted per kWh of electricity [S8], the electricity from this process can be converted to carbon dioxide emissions as follows.

(Amount of carbon dioxide converted from electricity [kmol/h]) = (Amount of carbon dioxide per kWh of electricity [kmol/kWh]) × (Power [kW])
= (0.855 × 0.454)/44.01 × 1045
= 9.22 kmol/h
Therefore, the amount of carbon dioxide emitted by this process is 136.1 + 83.6 + 9.22 = 228.9 kmol/h.

S2-2. Electrolysis of water by wind power (Electrolysis only, experimental)

Consider the carbon dioxide emissions of water electrolysis when using an electrolysis tank: as mentioned in Section S1-2, water electrolysis using an electrolysis tank produces 42 m^3/h of hydrogen gas with 200 kW of electricity [S1]. For the production of 228.7 kmol/h of hydrogen, from Section S1-2, it is 5.12×10^3 m^3/h, so the required electric power is obtained as follows:
(Electric power required [kW])
= (H_2 target production [m^3/h]) / (H_2 production [m^3/h] of commercial equipment) × (Electric power [kW] for commercial equipment)
= 5123 / 42 × 200 = 24395 kW
Hence, because wind power emits 11 g carbon dioxide per kWh [S9],
(Amount of carbon dioxide converted from electricity [kmol/h]) = (Amount of carbon dioxide per kWh of electricity [kmol/kWh]) × (Electricity [kW])
= 11×10^{-3} / 44.01 × 24395
= 6.097 kmol/h

S2-3. Electrolysis of water by wind power (Electrolysis only, theoretical)

Calculate the carbon dioxide emissions from the electrical energy required for the electrolysis of water by wind power as discussed in Section S1-3. From Section S1-3, assuming that the energy required is 441.2 GJ/h and the conversion efficiency η = 0.42 [S7], the carbon dioxide emissions can be calculated as follows:
(Carbon dioxide emissions [kmol/h]) = (Amount of carbon dioxide per kWh of electricity [kmol/kWh]) × (Electricity [kW])
= 11×10^{-3} / 44.01 × ($441.2\times0.2\times10^6$) / 3600
= 6.126 kmol/h

S2-4. Electrolysis of water by wind power (Reaction enthalpy)

The carbon dioxide emissions are calculated from the reaction of the enthalpy of water production. From Section S1-4, the energy required for H_2 production is 54.89 GJ/h. Therefore, the carbon dioxide emissions can be calculated as follows:

(Amount of carbon dioxide converted from electricity [kmol/h]) = $(11\times10^{-3} / 44.01) \times (54.89\times10^{6} / 3600)$

= 3.811 kmol/h

S2-5. Steam reforming of methane (w/ CO_2 capture)

Calculate the carbon dioxide emissions in the process of methane steam reforming with carbon dioxide capture. With carbon dioxide capture, 0.0888 kg of carbon dioxide is emitted per 1 Nm^3 of hydrogen in the process of methane steam reforming [S5], so the amount of carbon dioxide emitted to produce 41 million Nm^3 of hydrogen can be calculated.

(Amount of CO_2 emitted by the methane steam reforming process [kmol/h]) = (CO_2 emissions per Nm^3 of H_2 [kmol/(Nm^3)]) × (H_2 production [Nm^3/h])

= $0.0888 / 44.01 \times (4.1\times10^{7} / 8000)$

= 10.3 kmol/h

S2-6. Steam reforming of methane (w/o CO_2 capture)

Calculate the energy requirements for the methane steam reforming process without CO_2 capture. Without CO_2 capture, the methane steam reforming process emits 0.8091 kg/Nm^3 of CO_2 [S5], so the amount of CO_2 emitted to produce 228.7 kmol/h of H_2 can be calculated.

(Amount of CO_2 emitted by the methane steam reforming process [kmol/h]) = (CO_2 emission per Nm^3 of H_2 [kmol/(Nm^3)]) × (H_2 production [Nm^3/h])

= $0.8091 / 44.01 \times (4.1\times10^{7} / 8000)$

= 94.2 kmol/h

S2-7. Steam reforming of methane (Reaction enthalpy)

Calculate the CO_2 emission from the reaction in Eqs. S1-7-1 and S1-7-2. The amount of CO_2 emitted in the reaction process of steam reforming of methane with 228.7 kmol/h of H_2 can be calculated in the following way:

(Amount of CO_2 emitted by the steam reforming of methane [kmol/h]) = (molar ratio of H_2 to CO_2) × (H_2 production [kmol/h])
= 1/4 × 228.7
= 57.2 kmol/h
The amount of CO_2 emitted from the furnace used in the methane steam reforming process is then calculated. Calculated as in this process, the calculation is as follows.
(Amount of hexane required for the heating furnace [kmol/h]) = (Energy required by the heating furnace [GJ/h]) / ((Heat of combustion of hexane [GJ/kmol])
= 28.3 / (2031949×10^{-6})
= 1.72 kmol/h
Hence, since the molar ratio of hexane to CO_2 is six from the following reaction equation,
$2C_6H_{14} + 19O_2 \rightarrow 12CO_2 + 14H_2O$,
the amount of CO_2 emitted can be calculated as follows:
(Amount of CO_2 emitted from the furnace [kmol/h]) = (Amount of hexane burned [kmol/h]) × (Molar ratio of hexane to CO_2)
= 1.725 × 6
= 10.4 kmol/h
Therefore, the amount of CO_2 emitted in this process is 57.2 + 10.4 = 67.6 kmol/h.

S2-8. Electrolysis of water by electric power system (Electrolysis only, experimental)

From Section S2-2, the electric power required for the electrolysis of water is 24395 W. If 0.855 lb of CO_2 is emitted to generate 1 kWh of electricity [S8], the CO_2 emissions can be calculated as follows:
(Amount of CO_2 converted from electricity [kmol/h]) = (Amount of CO_2 per kWh of electricity [kmol/kWh]) × (Electricity [kW])
= (0.855 × 0.454) / 44.01 × 24395
= 215 kmol/h

S2-9. Electrolysis of water by electric power system (Electrolysis only, theoretical)

From Section S1-9, the CO_2 emission is calculated based on 210.1 GJ/h of energy required for the electrolysis of water. Assuming a conversion efficiency η = 0.42 [S7], the following can be calculated:
(CO_2 emission [kmol/h]) = (Amount of CO_2 per kWh of electricity [kmol/kWh]) ×

(Electricity [kW])
= (0.855 × 0.454) / 44.01 × 24510
= 216 kmol/h

S2-10. Electrolysis of water by electric power system (Reaction enthalpy)

Based on the enthalpy of water production from Section S1-4, the energy required for H_2 production is 54.89 GJ/h. Therefore, the carbon dioxide emissions can be calculated as follows:
(Amount of CO_2 converted from electricity [kmol/h]) = (Amount of CO_2 per kWh of electricity [kmol/kWh]) × (Electricity [kW])
= (0.855×0.454) / 44.01 × (54.89×10^6 / 3600)
= 134.5 kmol/h

S3. Economic Cost

S3-1. Decomposition of cellulose (This study)

From Section 3-8, the cost of our hydrogen production process to produce 228.7 kmol/h of hydrogen is $78.8M per year.

S3-2. Electrolysis of water by wind power (Electrolysis only, experimental)

When an electrolysis tank is used, the cost of water electrolysis can be calculated as follows, assuming CAPEX is $988/kW and OPEX is $40/kW [S10].
(Cost of water electrolysis [$]) = {(CAPEX per 1kW [$/kW]) + (OPEX per 1kW [$/kW])} × (Power [kW])
= (988 + 40) × 200 × 5123 / 42
= $25.1M

S3-3. Electrolysis of water by wind power (Electrolysis only, theoretical)

Find the cost of electrolysis of water as follows:
(Cost required for H_2 production [$]) = (Cost of raw material of water [$]) + (Cost of electrolysis [$])

$= 228.7 \times 8000 \times 18.02 \times 0.00022 + (210.1\times10^6 \times 8000 \times 0.1) / 3600$
= \$46.7M

S3-4. Electrolysis of water by wind power (Reaction enthalpy)

Also, calculate the cost of water calculated from the enthalpy of water production.
(Cost for H_2 production [\$]) = (Raw material cost of water [\$]) + (Cost of electrolysis [\$])
$= 228.7 \times 8000 \times 18.02 \times 0.00022 + (54.89\times10^6 \times 8000 \times 0.1) / 3600$
= \$17.7M

S3-5. Steam reforming of methane (w/ CO_2 capture)

For CO_2 absorption, the cost is 16.5 c€⁄(Nm^3) (0.18 \$/Nm^3)[S5], which can be calculated as follows.
(Cost for H_2 production [\$]) = (Water production [Nm^3]) × (LCOH [\$⁄(Nm^3)])
$= 4.1\times10^7 \times 16.5 \times 1.09\times10^{-2}$
= \$7.37M

S3-6. Steam reforming of methane (w/o CO_2 capture)

Consider the cost of the methane steam reforming reaction. Since the hydrogen produced in this process is 41 million Nm^3, the cost is 11.4 c€⁄(Nm^3) (0.12 \$⁄(Nm^3))[S5] without considering CO_2 absorption, which can be calculated as follows.
(Cost for H_2 production [\$]) = (Water production [Nm^3]) × (LCOH [\$⁄(Nm^3)])
$= 4.1\times10^7 \times 11.4 \times 1.09\times10^{-2}$
= \$5.09M

S3-7. Steam reforming of methane (Reaction enthalpy)

In terms of reactions, consider the cost of steam reforming of methane. Assuming that the feedstock cost of methane is 0.823 \$/Nm^3[S11], per Nm^3 of methane, we obtain:
(Cost required for H_2 production [\$]) = (Raw material cost of methane [\$]) + (Raw material cost of water [\$]) + (Furnace cost [\$]) + (Fuel cost of hexane [\$])

$= (228.7\times10^3 / 4 \times 8000) \times 0.0224 \times 0.823$
$+ (228.7\times10^3 / 2 \times 8000) \times 18.02 \times 0.00022$
$+ 0.1417\times10^6$
$+ 1.72 \times 86.18 \times 8000 \times 0.23$
= $8.8521M

S3-8. Electrolysis of water by electric power system (Electrolysis only, experimental)

Same as Section S3-2, assuming that the cost of electricity from wind power is the same as the cost of electricity from the power grid.

S3-9. Electrolysis of water by electric power system (Electrolysis only, theoretical)

Same as Section S3-3, assuming that the cost of electricity from wind power is the same as the cost of electricity from the power grid.

S3-10. Electrolysis of water by electric power system (Reaction enthalpy)

Same as Section S3-4, assuming that the cost of electricity from wind power is the same as the cost of electricity from the power grid.

References

[S1] Zeng, K.; Zhang, D. Recent progress in alkaline water electrolysis for hydrogen production and applications. *Prog. Energy Combustion Sci.* **2010**, *36*, 307–326. https://doi.org/10.1016/j.pecs.2009.11.002

[S2] https://www.epa.gov/sites/default/files/2019-08/documents/wind_turbines_fact_sheet_p100il8k.pdf

[S3] Rosen, M. A.; Naterer, G. F.; Chukwu, C. C.; Sadhankar, R.; Suppiah, S. Nuclear-based hydrogen production with a thermochemical copper–chlorine cycle and supercritical water reactor: equipment scale-up and process simulation. *Int. J. Energy Res.* **2012**, *36*, 456–465.

https://doi.org/10.1002/er.1702

[S4] Orhan, M. F.; Dinçer, I.; Rosen, M. A. Efficiency comparison of various design schemes for copper–chlorine (Cu–Cl) hydrogen production processes using Aspen Plus software. *Energy Conv. Manag.* **2012**, *63*, 70–86. https://doi.org/10.1016/j.enconman.2012.01.029

[S5] https://ieaghg.org/exco_docs/2017-02.pdf

[S6] Ding, Y.; Alpay, E. Adsorption-enhanced steam–methane reforming. *Chem. Eng. Sci.* **2000**, *55*, 3929–3940. https://doi.org/10.1016/S0009-2509(99)00597-7

[S7] https://www.tepco.co.jp/en/corpinfo/illustrated/electricity-supply/thermal-international-e.html

[S8] https://www.eia.gov/tools/faqs/faq.php?id=74&t=11

[S9] Dolan, S. L.; Heath, G. A. Life cycle greenhouse gas emissions of utility-scale wind power: Systematic review and harmonization. *J. Ind. Ecology* **2012**, *16*, S136–S154. https://doi.org/10.1111/j.1530-9290.2012.00464.x

[S10] https://theicct.org/wp-content/uploads/2021/06/final_icct2020_assessment_of-_hydrogen_production_costs-v2.pdf

[S11] https://www.meti.go.jp/shingikai/energy_environment/methanation_suishin/pdf/009_03_04.pdf (in Japanese)
